# Gene Duplication and Transference of Function in the paleo*AP3* Lineage of Floral Organ Identity Genes

**DOI:** 10.3389/fpls.2018.00334

**Published:** 2018-03-23

**Authors:** Kelsey D. Galimba, Jesús Martínez-Gómez, Verónica S. Di Stilio

**Affiliations:** Department of Biology, University of Washington, Seattle, WA, United States

**Keywords:** ABC model, MADS-box genes, B-class genes, ectopic petaloidy, flower development, non-core eudicot, VIGS, *Thalictrum thalictroides*

## Abstract

The floral organ identity gene *APETALA3* (*AP3*) is a MADS-box transcription factor involved in stamen and petal identity that belongs to the B-class of the ABC model of flower development. *Thalictrum* (Ranunculaceae), an emerging model in the non-core eudicots, has *AP3* homologs derived from both ancient and recent gene duplications. Prior work has shown that petals have been lost repeatedly and independently in Ranunculaceae in correlation with the loss of a specific *AP3* paralog, and *Thalictrum* represents one of these instances. The main goal of this study was to conduct a functional analysis of the three *AP3* orthologs present in *Thalictrum thalictroides*, representing the paleo*AP3* gene lineage, to determine the degree of redundancy versus divergence after gene duplication. Because *Thalictrum* lacks petals, and has lost the petal-specific *AP3*, we also asked whether heterotopic expression of the remaining *AP3* genes contributes to the partial transference of petal function to the first whorl found in insect-pollinated species. To address these questions, we undertook functional characterization by virus-induced gene silencing (VIGS), protein–protein interaction and binding site analyses. Our results illustrate partial redundancy among *Thalictrum AP3*s, with deep conservation of B-class function in stamen identity and a novel role in ectopic petaloidy of sepals. Certain aspects of petal function of the lost *AP3* locus have apparently been transferred to the other paralogs. A novel result is that the protein products interact not only with each other, but also as homodimers. Evidence presented here also suggests that expression of the different *ThtAP3* paralogs is tightly integrated, with an apparent disruption of B function homeostasis upon silencing of one of the paralogs that codes for a truncated protein. To explain this result, we propose two testable alternative scenarios: that the truncated protein is a dominant negative mutant or that there is a compensational response as part of a back-up circuit. The evidence for promiscuous protein–protein interactions via yeast two-hybrid combined with the detection of AP3 specific binding motifs in all B-class gene promoters provide partial support for these hypotheses.

## Introduction

Gene duplication has long been interpreted as a potential source of raw genetic material acted upon by evolution (Ohno, 1970; Soukup, 1974; Force et al., 1999). Genes in the ABC model of flower development have been an especially targeted, undergoing multiple duplication events during the course of angiosperm evolution (Theissen et al., 1996; Airoldi and Davies, 2012). Because changes to floral organ identity genes have a profound effect on flower development, they present an ideal system to study the outcome of gene duplication in relation to morphological adaptation (Soltis and Soltis, 2014).

In the model angiosperm *Arabidopsis thaliana*, the B-class consists of two members, *APETALA3* (*AP3*) and *PISTILLATA* (*PI*). Both are necessary in combination with the E-class genes *SEPALLATA1–4* (*SEP1–4*), for petal identity in the second and stamen identity in the third whorl (Bowman et al., 1989, 1991; Coen and Meyerowitz, 1991; Theißen and Saedler, 2001). When B-class function is lost, stamen primordia are homeotically converted into carpels and petal primordia into sepals (Krizek and Meyerowitz, 1996). B-class gene function is highly conserved throughout angiosperms, occurring in core and non-core eudicots as well as monocots (Kim et al., 2004; Zahn et al., 2005; Litt and Kramer, 2010; Di Stilio, 2011). Nevertheless, there are examples of B-class genes being expressed in other plant organs, e.g., in root nodules of alfalfa (Heard and Dunn, 1995) and in first whorl petaloid tepals of tulips (Kanno et al., 2003), suggesting that they may have adopted novel roles in several lineages.

**FIGURE 1:**
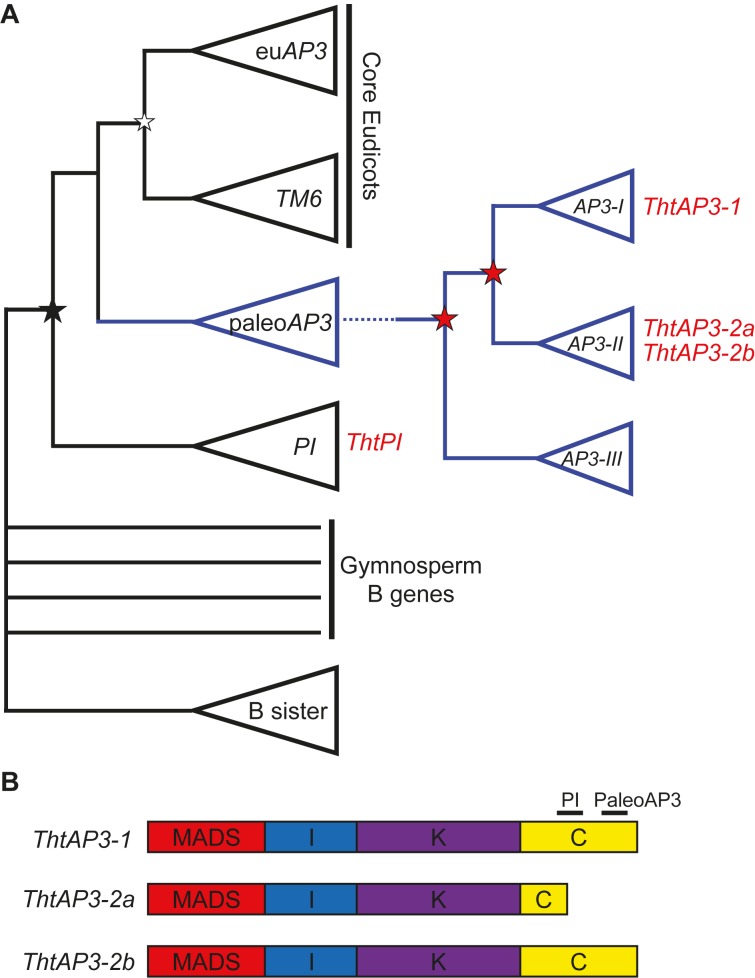
Simplified B-class gene phylogeny illustrating the position of *Thalictrum thalictroides* loci (in red) and the comparative structure of their protein products. **(A)** Simplified phylogeny, modified from Jaramillo and Kramer (2007) and Sharma et al. (2011). An ancient duplication in the angiosperm stem group led to the *AP3* and *PI* lineages (black star), the latter with one representative from *T. thalictroides* (*ThtPI*). In the core eudicots, a more recent duplication led to the eu*AP3* (containing the original *APETALA3*) and *TM6* lineages (white star). In the paleo*AP3* lineage, two Ranunculales-wide duplication events (red stars) led to the three paralogous clades *AP3-I* (including *ThtAP3-1*), *AP3-II* (including the more recently duplicated *ThtAP3-2a* and *ThtAP3-2b*), and *AP3-III* (no *AP3-III* genes have been recovered from *Thalictrum*). **(B)** MIKC structure of the three ThtAP3 protein products. ThtAP3-2a is truncated by an early stop codon, resulting in the loss of 18 amino acids, including the PaleoAP3 motif and a portion of the PI motif (Di Stilio et al., 2005).

After an ancient duplication leading to the *AP3* and *PI* lineages, in the stem group of the order Ranunculales, two duplication events led to three paralogous lineages of *AP3*: *AP3-I*, *AP3-II*, and *AP3-III* (Kramer et al., 2003; **Figure 1A**). *Thalictrum thalictroides* (Ranunculaceae), a member of the Ranunculid lineage that is sister to all other eudicots (Soltis et al., 2011; Maia et al., 2014), has one *PI* (*ThtPI*) and three *AP3* orthologs (*ThtAP3-1*, *ThtAP3-2a*, and *ThtAP3-2b*) (Kramer et al., 2003; Di Stilio et al., 2005). The paralog *ThtAP3-1* belongs to the *AP3-I* clade, while *ThtAP3-2a* and *ThtAP3-2b* belong to the *AP3-II* clade and are products of a more recent duplication that likely occurred in the common ancestor of *Thalictrum* and *Aquilegia* (Sharma et al., 2011). *ThtAP3-2a* contains a premature stop, resulting in a truncation affecting the conserved C-terminal motif of the protein; 18 residues encompassing half of the “PI motif-derived” and all the “paleoAP3” motif is missing (**Figure 1B**; Di Stilio et al., 2005). No *AP3-III* paralog has been identified so far in *Thalictrum* by PCR (Di Stilio et al., 2005; Zhang et al., 2013) or by BLAST search of available transcriptomes (unpublished data). Functional studies of the *Aquilegia coerulea AP3-III* ortholog provide evidence for sub-functionalization of this gene to petal identity (Sharma et al., 2011) and this function appears to be conserved throughout the order, including Papaveraceae (Arango-Ocampo et al., 2016); the lack of an *AP3-III* ortholog in *Thalictrum* therefore correlates with the loss of petals in the genus (Di Stilio et al., 2005; Zhang et al., 2013).

Petals may be interpreted as adaptive structures that attract biotic pollinators and this function has been transferred, fully or in part, to other organ types multiple times during angiosperm evolution (Cronk et al., 2002). In fact, in the Ranunculaceae, where sepals are often petaloid, petals have been lost independently multiple times with the role of attraction adopted instead by showy sepals (Zhang et al., 2013). In a number of distantly related species, including *Aristolochia* and *Tulipa*, heterotopic expression of B-class genes has been proposed as causative of petaloidy of first-whorl organs (van Tunen et al., 1993; Kanno et al., 2003; Jaramillo and Kramer, 2004). In *Aquilegia*, *AqAP3*s are not involved in petal identity or development of papillate epidermal cells, although they do contribute to the production of anthocyanin in petaloid sepals (Jaramillo and Kramer, 2007; Sharma et al., 2011; Sharma and Kramer, 2017). Although *T. thalictroides* flowers lack petals from inception, its sepals have a number of features typically associated with petals: they are non-photosynthetic (white or pink), relatively large (surpassing stamens), they may contain papillate cells in the upper epidermis (Di Stilio et al., 2009) and they express all B-class genes (Di Stilio et al., 2005). Sepals of wind pollinated species such as *Thalictrum dioicum*, on the other hand, are smaller (shorter than stamens), green, lack papillate cells, and do not express *AP3* orthologs (Di Stilio et al., 2005; Soza et al., 2012).

The goal of this study was to conduct a functional analysis of the three *AP3* orthologs in *T. thalictroides*, a ranunculid with representatives of the paleo*AP3* lineage of B-class genes. We aimed to determine the degree of redundancy versus divergence amongst these paralogs, while comparing their role to the more widely characterized eu*AP3* lineage. Finally, we investigated whether one or more of these loci are involved in ectopic petaloidy, in the form of partial transference of petal function to sepals, in the insect-pollinated species *T. thalictroides*. We addressed these questions via targeted silencing of individual genes, by determining the ability of each protein product to dimerize and by identifying AP3 binding sites within promoter regions of all four B-class genes.

## Materials and Methods

### Gene Sequences

Partial coding sequences for *ThtAP3-1*, *ThtAP3-2a*, and *ThtAP3-2b* were obtained from GenBank (AY162886, AY162887, and AY162888; Kramer et al., 2003). Full coding sequences were obtained from a 1KP *T. thalictroides* transcriptome^1^. Full genomic sequences and promoter regions were obtained from a *T. thalictroides* reference genome (unpublished) and deposited in GenBank (MG889397, MG889396, and MG889395).

### Plant Materials

*Thalictrum thalictroides* bare root plants were purchased from nurseries and grown in the University of Washington (UW) Greenhouse under ambient conditions from mid-February through mid-May. A voucher specimen is deposited at the UW Herbarium (V. Di Stilio 123, WTU 376542).

### Virus-Induced Gene Silencing

Regions for targeted gene silencing of *Thalictrum AP3* genes were selected to exclude areas with more than 13 continuous homologous base pairs among the three paralogs (gene duplicates). Fragments were amplified by PCR from a representative clone using primers with added *Bam*HI and *Kpn*I restriction sites (Supplementary Table S1), ligated to linearized tobacco rattle virus vector (TRV2; Liu et al., 2002) and transformed into *Agrobacterium tumefaciens* strain GV3101. The TRV2-*ThtAP3-1* construct was prepared using a 429 bp fragment comprising the C-terminal region (282 bp) and 3′UTR (147 bp) of *ThtAP3-1*. The TRV2-*ThtAP3-2a* construct consisted of a 427 bp fragment comprising the C-terminal region (227 bp) and 3′UTR (200 bp) of *ThtAP3-2a.* The TRV2-*ThtAP3-2b* construct consisted of a 408 bp fragment comprising the C-terminal region (253 bp) and 3′UTR (155 bp) of *ThtAP3-2b*. Additional experiments targeting shorter regions (216–342 bp) were attempted first, but they did not trigger sufficient down-regulation. Experiments using double and triple constructs were also attempted unsuccessfully.

Twenty *T. thalictroides* tubers that had been kept in soil in the dark at 4°C for 8 weeks, were treated with each construct as described previously (Di Stilio et al., 2010; Galimba et al., 2012). Briefly, a small incision was cut in the tubers near the bud using a clean razor blade, they were then submerged in infiltration medium containing appropriate *Agrobacterium* cultures (TRV1 and one of the TRV2 constructs), and infiltrated in a chamber under full vacuum (-100 kPa) for 10 min. Nine TRV2-*ThtAP3-1*, five TRV2-*ThtAP3-2a*, and 12 TRV2-*ThtAP3-2b* treated plants survived to flowering. Twenty plants were infiltrated with empty TRV2 vector (mock-treated control, to detect background viral effects). All plants were transferred to the greenhouse following infiltration and were grown together with 20 untreated plants under equal conditions. Leaves and flowers arose approximately 2 weeks later. Flowers from all treatments and controls (untreated and treated with empty vector) were observed, as they opened, under a Nikon SMZ800 dissecting scope, photographed using a Q-Imaging MicroPublisher 3.3 digital camera or a Canon PowerShot SD890 IS digital camera, and flash-frozen for RNA processing.

### Molecular Validation of VIGS Lines

After recording their phenotype and photographing, young open flowers that had been flash frozen (as described above) were processed to determine the presence of TRV1 and TRV2 constructs, and the expression levels of B-class genes, including the three *Thalictrum AP3* genes and the single copy gene *ThtPI*. Total RNA was extracted using TRIzol (Invitrogen, Life Technologies, CA, United States), following manufacturer instructions and DNased using amplification grade DNase (Invitrogen, Life Technologies, CA, United States). First strand cDNA synthesis was carried out on 1 μg of total mRNA using iScript (Bio-Rad, CA, United States), following manufacturer instructions, and diluted 2.5-fold for use in quantitative reverse transcriptase (RT) PCR (qPCR). PCR was carried out with GoTaq (Promega, WI, United States) on 1 μl of cDNA using TRV1- and TRV2-specific primers (Supplementary Table S1) for 30 cycles at 51°C annealing temperature. Locus-specific primers, designed to amplify regions distinct from those used in the virus-induced gene silencing (VIGS) constructs, were used to test expression levels of *ThtAP3-1*, *ThtAP3-2a*, *ThtAP3-2b*, and *ThtPI* by qPCR (Supplementary Table S1). Primers were validated prior to use, using a cDNA dilution series to verify consistent efficiencies and a dissociation curve analysis to confirm single product amplification, as previously published (Galimba et al., 2012). Each qPCR reaction contained 15 μl iQ SYBR Green Supermix (Bio-Rad, CA, United States), 12.2 μl H_2_O, 0.9 μl each of forward and reverse primer and 1 μl of cDNA. Samples were amplified on an MJ Research Chromo4 Detector for 45 cycles, in triplicate, including a no-template control. Cycling conditions were: 94°C for 10 min and 45 cycles of 94°C for 30 s, 53°C for 30 s, and 72°C for 30 s. Relative expression was calculated using the 2^-ΔΔ*Ct*^ method (Livak and Schmittgen, 2001), and normalized against the averaged expression of two housekeeping genes, *ThtACTIN* and *ThtEF1*α (*Elongation Factor 1*α). Since B-class gene expression levels between untreated (*n* = 6) and empty TRV2-treated flowers (*n* = 3) were not significantly different in a Student’s two-tailed *t*-test with unequal variance (*p* > 0.05), both controls were combined for further analyses. The statistical significance of differences in gene expression between controls and treatments was calculated using Student’s two-tailed *t*-test with unequal variance: controls (*n* = 9), TRV2-*ThtAP3-1* (*n* = 15), TRV2-*ThtAP3-2a* (*n* = 10), and TRV2-*ThtAP3-2b* (*n* = 4), and *p*-values were evaluated using Holm–Bonferroni corrections to avoid Type I error.

### Yeast Two-Hybrid Assays

To prepare constructs for yeast two-hybrid analysis, complete coding sequences for ThtAP3-1, ThtAP3-2a, and ThtAP3-2b were cloned into pGADT7 and pGBKT7 vectors using the In-Fusion HD Cloning Kit (Clontech, CA, United States) and custom primers (Supplementary Table S1). The pGADT7 and pGBKT7 constructs for the E-class interacting partner ThtSEP3ΔC were available from a previous study (Galimba et al., 2012). ThtSEP3ΔC is a C-terminal-truncated version of ThtSEP3 lacking the last 195 bp of coding sequence to avoid auto-activation (Causier and Davies, 2002; Galimba et al., 2012); C-terminal truncation is supported by a number of studies showing that the K domain is more critical for MADS-box protein interactions (Davies et al., 1996; Pelaz et al., 2001; Immink et al., 2009) and is involved both in dimer and tetramer formation (Puranik et al., 2014).

Yeast two-hybrid assays were carried out using Matchmaker Gold Yeast Two-Hybrid System (Clontech, CA, United States). Cells were co-transformed and plated on Leu/Trp-free media to select for diploid colonies. Single colonies were serially diluted 10-fold to 1:10,000 in water and 5 μl of each dilution were plated on Leu/Trp/His-free media and Leu/Trp/His-free media supplemented with Aureobasidin A (AbA) to test for protein interaction, and on Leu/Trp-free media to control for yeast growth. Interactions were scored and photographed using a Canon PowerShot SD890 IS digital camera after 4 days of incubation at 28°C.

### Identification of Promoters and *APETALA3* Binding Motif Analysis

In *A. thaliana*, the region extending 496 bp upstream of the *AP3* start codon contains three CArG boxes and drives GUS expression in the same temporal and spatial patterns as the *AP3* transcript in wild-type flowers (Tilly et al., 1998). Using this information as guideline, and to identify promoters in *Thalictrum AP3*, a 1 kb fragment upstream of the start codon of each gene was obtained from a *T. thalictroides* draft genome (unpublished). The *Arabidopsis AP3* frequency matrix was downloaded from JASPAR^2^ (Mathelier et al., 2014; **Figure 5A**). This matrix, derived from ChIP-seq data (Wuest et al., 2012), consists of nucleotide frequencies at 15 positions: a 10-nucleotide CArG box surrounded by two 5′ and three 3′ nucleotides. The use of frequency matrices provides position-specific penalties for deviations from the consensus as opposed to simpler models, like consensus sequences, which treat all mismatches equally (Stormo, 2013). We converted the matrix to a position-specific scoring matrix (PSSM) using the RSAT-convert-matrix tool available at Regulatory Sequence Analysis Tools^3^ (Medina-Rivera et al., 2015). The *A. thaliana* background model estimation method was utilized with default settings. The PSSM was used in MORPHEUS^4^ (Minguet et al., 2015) to search sections of sequence upstream of the START codon of *TthAP3-1*, *ThtAP3-2a*, *ThtAP3-2b*, and *ThtPI* for AP3 binding sites. MORPHEUS provides a score for each binding site that is based on the relative affinity of the binding site to the provided matrix, the threshold was set to 5 based on the score distribution histogram (Supplementary Figure S2) to limit the results to the best matching sites.

To visualize the degree of conservation of the *T. thalictroides* putative AP3 binding motifs (found by the method described above), we generated two alignments of the first 500 bp upstream of the start codon of paleo*AP3-1* and paleo*AP3-2* orthologs of other available Ranunculaceae. Sequences were retrieved from GenBank and Phytozome (Supplementary Table S2 and Supplementary Figure S3), aligned using MUSCLE (Edgar, 2004) and CLC Main Workbench 7 (Qiagen), and homologous regions to the *T. thalictroides* AP3 binding sites were identified.

### Analysis of Protein Structure

In order to identify the alpha helices known to be critical for MADS box gene functionality (Puranik et al., 2014), and to test whether any of these were lacking in the paralog coding for a truncated AP3 (*ThtAP3-2a*), we analyzed the protein structure of the region comprising the end of the I domain through the C domain of the three *Thalictrum* AP3 proteins using Protein Homology/analogY Recognition Engine v 2.0 (Phyre^2^; Kelley et al., 2015).

## Results

### Targeted Gene Silencing of Three *T. thalictroides AP3* Orthologs

To investigate the function of three B-class gene orthologs of the ranunculid *T. thalictroides*, we conducted targeted VIGS using single gene constructs, analyzed gene expression of treated plants in relation to untreated and empty vector controls, and compared the resulting floral phenotypes.

**FIGURE 2:**
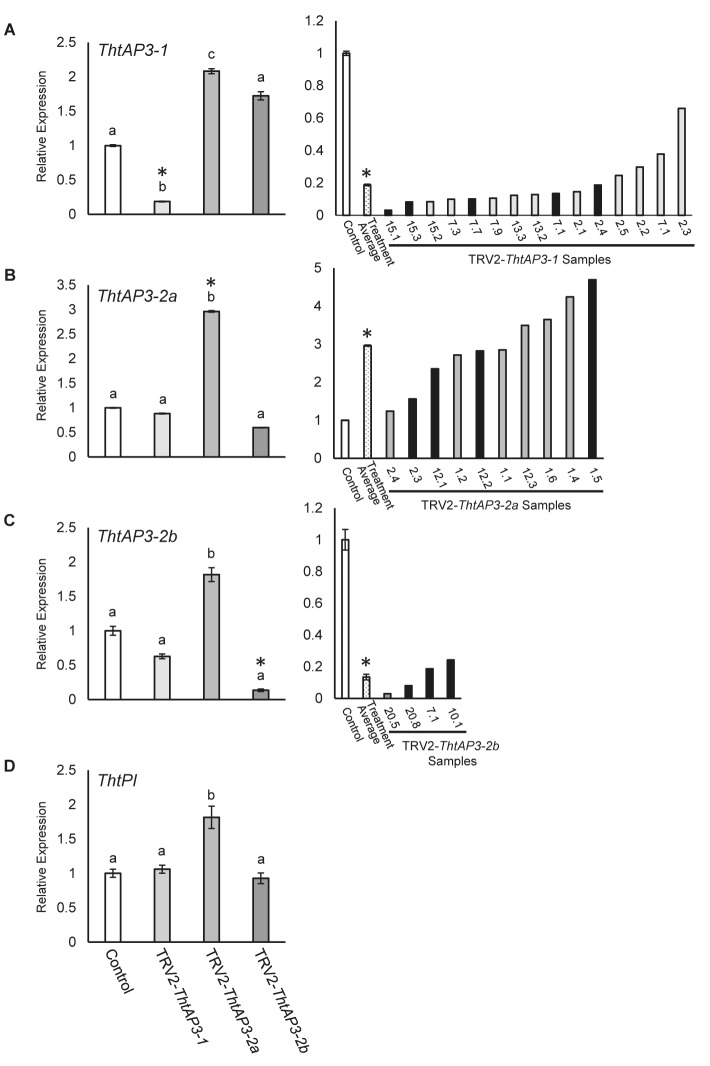
Molecular validation of targeted gene silencing of *Thalictrum thalictroides AP3* orthologs. **(A–D)** Left panels: Quantitative RT-PCR (qPCR) showing average relative expression levels of *ThtAP3-1* **(A)**, *ThtAP3-2a* **(B)**, *ThtAP3-2b* **(C)**, and *ThtP1* **(D)** in each of three treatments, TRV2-*ThtAP3-1*, TRV2-*ThtAP3-2a*, and TRV2-*ThtAP3-2b* by virus-induced gene silencing. Control (*n* = 9: untreated, *n* = 6 plus TRV2-empty, *n* = 3); *ThtAP3-1* VIGS (*n* = 15); *ThtAP3-2a* VIGS (*n* = 10); and *ThtAP3-2b* VIGS (*n* = 4). Gene expression levels of the control were set to 1. Expression was normalized against *ThtACTIN* and *ThtEEF1*. Mean and SE of biological replicates are shown; different letters indicate a significant difference in Student’s two-tailed *t*-test with unequal variance and Holm–Bonferroni corrections (*p* ≤ 0.05). **(A–C)** Right panels: Molecular validation of targeted gene silencing in individual flowers (labels represent plant number, followed by flower number) relative to control (set to 1). Treatment average (dotted bar) of individual flowers (biological replicates). SE shown for control and treatment average values. Black bars indicate flowers shown in **Figure 3**. Asterisks cross-reference same treatment averages compared between treatments (left), or amongst individual samples within a treatment (right).

**FIGURE 3:**
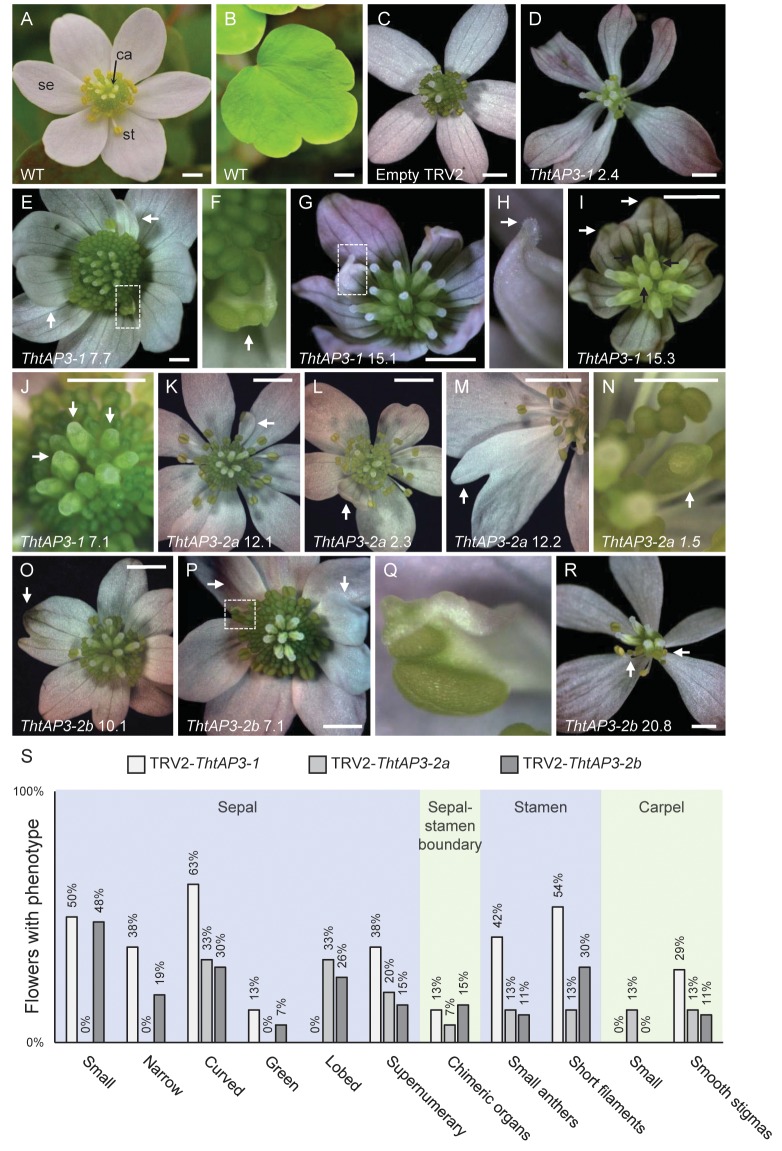
Phenotypes resulting from targeted silencing of *Thalictrum thalictroides* AP3 orthologs. **(A)** Untreated *T. thalictroides* representative flower. **(B)** Untreated representative leaflet. **(C)** Flower treated and validated for empty TRV2 vector. **(D–J)** Representative flowers treated with and validated for TRV2-*ThtAP3-1*. **(D)** Flower with narrow sepals. **(E)** Flower with chimeric organ (boxed) and extra inner sepals (arrows). **(F)** Detail of sepal/stamen chimera in panel **(E)** (arrow). **(G)** Flower with small, curved sepals and chimeric organ in place of outer stamen (boxed). **(H)** Detail of sepal–carpel chimera in panel **(G)**, with stigma (arrow). **(I)** Flower with small, curved, greener sepals, green segments indicated by white arrows, and smooth stigmas, indicated with black arrows. **(J)** Flower with smooth stigmas, three indicated with arrows. **(K–N)** Representative flowers treated with TRV2-*ThtAP3-2a*. **(K)** Flower with chimeric sepal/stamen organ in place of outer stamen (arrow). **(L)** Flower with extra inner sepal (arrow). **(M)** Flower with lobed sepal (arrow). **(N)** Flower with unfused carpel and exposed ovule (arrow). **(O–R)** Representative flowers treated with TRV2-*ThtAP3-2b*. **(O)** Flower with lobed sepal with green sectors (arrow). **(P)** Flower with chimeric organ (boxed) and extra inner sepals (arrows). **(Q)** Detail of sepal/stamen chimera in panel **(O)**. **(R)** Flower with narrow sepals and stunted stamens exhibiting small anthers and short filaments (arrows). **(S)** Percentage of flowers exhibiting each phenotype (from plants verified for TRV1/2 transcripts): TRV2-*ThtAP3-1* (*n* = 24); TRV2-*ThtAP3-2a* (*n* = 15); and TRV2-*ThtAP3-2b* (*n* = 27). Sample labels represent plant number, followed by flower number (see **Figure 2** for individual expression levels of target genes). Scale bar = 1 mm.

Flowers treated with empty TRV2 vectors and verified for the presence of TRV1/2 transcripts (Supplementary Figure S1) were combined with untreated wild-type flowers, and used as a control treatment for molecular validation (*n* = 9 flowers from four plants; **Figure 2**). Wild-type *T. thalictroides* flowers are apetalous, with 5–12 white or pink petaloid sepals enclosing 45–76 filamentous stamens and 3–11 free carpels with prominent papillae at the stigma (Di Stilio et al., 2009; Galimba et al., 2012; **Figure 3A**). Wild-type leaves are compound with lobed leaflets (**Figure 3B**). Empty TRV2-treated flowers showed comparable morphology to wild-type (**Figure 3C**). Small sections of brown necrotic tissue were present on 19% of treated flowers, similar to previously reported background viral effect (Di Stilio et al., 2010), and these were therefore not counted as phenotype.

Flowers treated with TRV2-*ThtAP3-1* and verified for the presence of TRV1/2 transcripts (*n* = 15 flowers from four plants; Supplementary Figure S1) exhibited a significant down-regulation of *ThtAP3-1* transcripts, with an average 5.3-fold decrease in gene expression levels with respect to controls (*p* < 0.001, **Figure 2A**). Down-regulation of *ThtAP3-1* at the individual level ranged from a 1.5- to 32-fold decrease in gene expression (**Figure 2A**). Average expression levels of the other three B-class genes were statistically like controls (**Figures 2B–D** and Supplementary Table S3). All flowers (*n* = 24) from validated plants were phenotyped (**Figure 3S**). Abnormal phenotypes included narrow sepals in 38% of flowers (**Figure 3D**), smaller and possibly extra inner sepals, against the outer stamens in 38% of flowers (**Figure 3E**, sepal number is variable in *Thalictrum*, but positioning and size of these is distinct) and chimeric organs (sepal/stamen or sepal/carpel) in the sepal/stamen boundary in 13% of flowers (**Figures 3E–H**). These chimeric organs included sepaloid organs with anther sacs (**Figures 3E,F**) or sepaloid organs with stigma-like tissue on the distal end (**Figures 3G,H**). We also observed stunted stamens with small anthers in 42% of flowers and/or short filaments in 54% of flowers (**Figures 3D,G,I**). Stunted stamens were not present in our empty TRV2-treated flowers, although we have observed them previously at low frequencies in viral controls (Soza et al., 2016). TRV2-*ThtAP3-1* treated plants also possessed small sepals on 50% of flowers and/or curved sepals on 63% of flowers (**Figures 3G,I**). We also observed patches of green tissue on sepals of 13% of flowers (**Figure 3I**) and smooth stigmas, due to the absence of stigmatic papillae, on 29% of flowers (**Figures 3I,J**). In summary, in addition to the expected role in stamen identity, down-regulation of *ThtAP3-1* altered sepal size (and possibly number), shape and color, changed organ identity at the sepal/stamen boundary and resulted in loss of stigmatic papillae.

Treatment with TRV2-*ThtAP3-2a* failed to down-regulate *ThtAP3-2a* in flowers verified for the presence of TRV1 and TRV2 transcripts (*n* = 10 flowers from three plants; Supplementary Figure S1). On the contrary, it resulted in up-regulation of all *ThtAP3s* and *ThtPI* (**Figures 2A–D**). These flowers showed, on average, a 2.9-fold increase in *ThtAP3-2a* expression, which was significantly higher than controls (*p* < 0.001; **Figure 2B**). Up-regulation of *ThtAP3-2a* at the individual level ranged from a 1.2- to 4.7-fold increase in gene expression (**Figure 2B**). They also had, on average, significantly higher gene expression levels than controls for *ThtAP3-1* (2.1-fold, *p* = 0.005; **Figure 2A** and Supplementary Table S3) and *ThtPI* (1.8-fold, *p* = 0.013; **Figure 2D** and Supplementary Table S3). *ThtAP3-2b* expression levels were 1.8-fold higher in TRV2-*ThtAP3-2a* treated flowers; this increase was not significant when applying Holm–Bonferroni corrections (*p* = 0.026; **Figure 2C** and Supplementary Table S3). Flower (*n* = 15) phenotypes from validated plants consisted of a subset of those observed in *ThtAP3-1* treated plants (**Figure 3S**). Sepal/stamen chimeric organs in place of outer stamens were present on 7% of flowers (**Figure 3K**), and small extra inner sepals were present on 20% (**Figure 3L**). Lobed sepals were present on 33% of treated flowers (**Figure 3M**) and were not present in any of the untreated plants. We observed curved sepals on 33% of flowers and stunted stamens with short filaments and/or small anthers on 13% of flowers. We did not observe narrow, small, or green outer sepals in this treatment. Small, underdeveloped carpels with smooth stigmas, including one with an exposed ovule (**Figure 3N**), were present in 13% of flowers. In summary, we were unable to detect down-regulation of *ThtAP3-2a* after treatment with TRV2-*ThtAP3-2a*, and instead detected unexpected up-regulation of all B-class genes (albeit not statistically significant for *ThtAP3-2b*), which was associated with phenotypes affecting stamen/sepal boundary, sepal morphology, and carpel development.

Flowers treated with TRV2-*ThtAP3-2b* and verified for the presence of viral transcripts (*n* = 4 flowers from three plants; Supplementary Figure S1) showed, on average, a strongly significant down-regulation of *ThtAP3-2b* compared to controls (2.8-fold decreased expression, *p* = 0.001; **Figure 2C**). *ThtAP3-2b* was down-regulated a 4.1- to 7.4-fold at the individual level (**Figure 2C**). All other B-class gene expression levels were like controls (**Figures 2A,B,D** and Supplementary Table S3). Flowers (*n* = 27) from validated plants (**Figure 3S**) showed lobed sepals on 26% of flowers, with green sectors on 7% of flowers (**Figure 3O**). Small sepals were also present on 48% of flowers and curved sepals were present on 30%. We also observed stamens replaced with chimeric organs (sepaloid with carpeloid or stamenoid features) on 15% (**Figures 3P,Q**) and small extra inner sepals on 15% of flowers (**Figure 3P**). Plants also displayed narrow sepals on 19% of flowers (**Figure 3R**) and stunted stamens, with short filaments on 30% of flowers and/or small anthers on 11% (**Figure 3R**). Smooth stigmas with reduced papillae were present on 4% of flowers. In summary, down-regulation of *ThtAP3-2b* altered sepal size, shape and color, stamen identity, sepal/stamen boundary, and stigmatic papillae development in a similar manner as the down-regulation of *ThtAP3-1*, with the addition of sepal lobing.

Taken together, phenotypes resulting from VIGS treatments (and not observed in control flowers) affected either sepal, stamen, or carpel morphology. Abnormal sepal morphology occurred in flowers across treatments: down-regulation of *ThtAP3-1* and *ThtAP3-2b* resulted in reduction in overall size and width (small and narrow), shape (curved instead of flat) and color (green), with *ThtAP3-2b* VIGS flowers also exhibiting sepal lobing. Similar lobing and curving of sepals, was observed in TRV2-*ThtAP3-2a* treated flowers, which exhibited higher expression of all B-class genes, although none of the additional sepal defects were present (narrower, smaller, or green). The second major category of phenotypes affected stamen morphology across the three treatments: stamens looked stunted, had small anthers, their filaments failed to properly elongate, and outer stamens were sometimes replaced by chimeric organs or small sepals. Lastly, down-regulation of *ThtAP3-1* and *ThtAP3-2b* affected late carpel development, leading to the absence of stigmatic papillae, while the high expression of B-class genes in TRV2-*ThtAP3-2a* treated flowers caused carpel stunting and failure to fuse around the ovule.

### Yeast Two-Hybrid Analyses: Promiscuous Interactions Among *Thalictrum* B-Class Proteins

**FIGURE 4:**
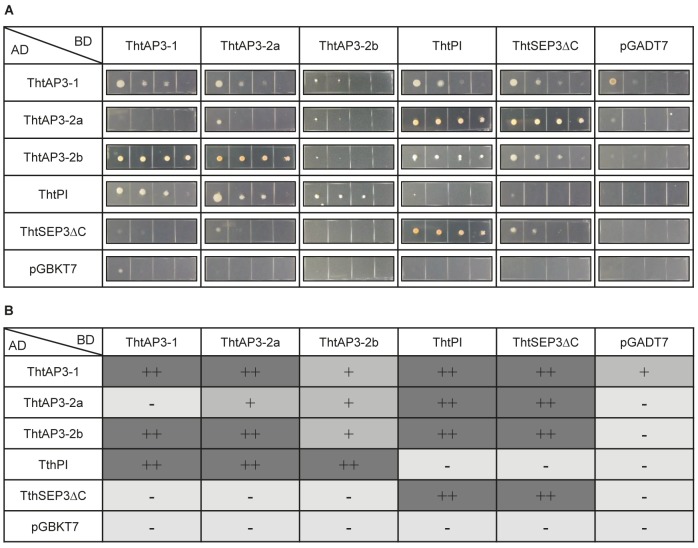
Protein–protein interactions among B-class gene products of the ranunculid *Thalictrum thalictroides*. Interactions between the three ThtAP3 proteins, ThtPI and ThtSEP3 of *T. thalictroides* were determined with the yeast two-hybrid system. The C-terminus of ThtSEP3 was truncated to avoid autoactivation (ThtSEP3ΔC). **(A)** Colony growth on selective Leu/Trp/His-free + AbA medium. Yeast cells were spotted in four 10-fold serial dilutions (from left to right) for each interaction tested. All proteins were expressed as fusion with the GAL4 activation domain (AD) and the GAL4 DNA binding domain (BD). pGBKT7 and pGADT7 are empty vector controls. **(B)** Interpretation of the strength of the protein–protein interactions shown in panel **(A)**. –, no interaction; +, interaction; ++, strong interaction.

To test the interactions among the four B-class proteins present in *T. thalictroides*, we performed yeast two-hybrid analyses, including the predicted E-class partner protein ThtSEP3 (**Figure 4**). We used a ThtSEP3ΔC construct with a truncated C-terminus to avoid previously documented auto-activation (Galimba et al., 2012). Control transformations with empty vectors (pGBKT7 and pGADT7) produced minimal to no growth, ruling out auto-activation for the other proteins.

As expected, all three ThtAP3 proteins could dimerize with their B-class partner ThtPI, and this interaction was observed in both directions. A more unexpected result was that all three ThtAP3 proteins could heterodimerize with each other: ThtAP3-1 interacted with ThtAP3-2a and ThtAP3-2b, and ThtAP3-2a interacted with ThtAP3-2b. These interactions varied in strength, and ThtAP3-1 and ThtAP3-2a had a positive interaction in one direction and a negative one in the other. ThtAP3-1 interacted weakly with the empty vector in one direction, and this may decrease support for the weak interaction detected between ThtAP3-1 and ThtAP3-2b. Using the yeast culture dilutions (**Figure 4A**) to interpret the strength of the protein–protein interactions (**Figure 4B**), we observed that ThtAP3-1 could homodimerize strongly, while ThtAP3-2a and ThtAP3-2b homodimerized weakly and ThtPI did not form homodimers. All four B-class proteins (ThtAP3-1, ThtAP3-2a, ThtAP3-2b, and ThtPI) could interact with the E-class partner ThtSEP3. Taken together, all three ThtAP3 proteins heterodimerize with ThtPI and ThtSEP3, as expected, and additionally homodimerize and heterodimerize with each other.

### Identification of Putative AP3 Binding Motifs in *T. thalictroides* B-Class Gene Promoters

**FIGURE 5:**
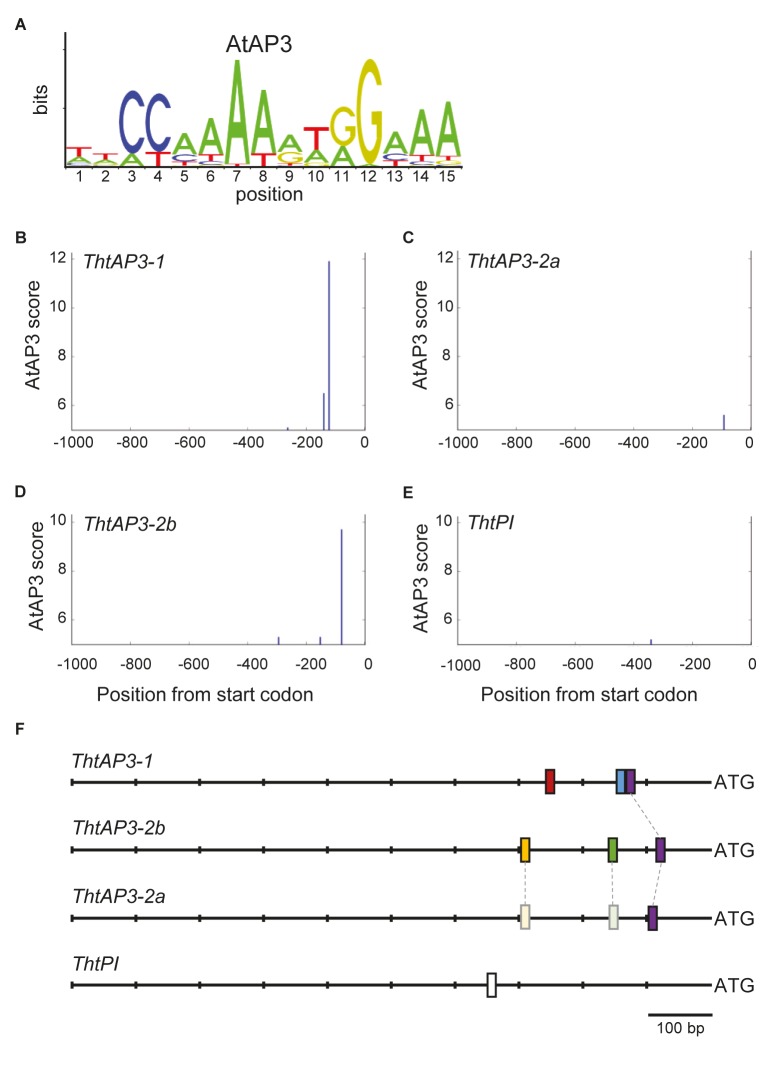
Location of APETALA3-like binding sites on *Thalictrum thalictroides* B-class gene promoters. **(A)** Sequence logo for *Arabidopsis* AP3 binding frequency matrix (jaspar.genereg.net), with letter sizes relative to nucleotide frequency. **(B–E)** Positions identified by MORPHEUS as AP3 binding sites, in 1 kb sections of sequence directly upstream of each gene. The height of each vertical bar indicates the MORPHEUS score of that binding site, with the cutoff score set at 5. **(F)** Schematics of promoter regions for each gene, with binding site positions indicated by bars. A putative homologous binding site shared by all three ThtAP3 promoters is shown in the same color (purple), and two putative homologous regions in ThtAP3-2a are shown in lighter shades (see also Supplementary Figure S3).

To find additional evidence for auto- and/or cross-regulation of B-class genes by ThtAP3 proteins, we searched for AP3 binding sites in *Thalictrum* B-class gene promoter regions using a frequency matrix (**Figure 5A**) derived from experimental binding assays that includes AP3-specific binding frequencies to the CArG box plus five additional nucleotides (Sharma et al., 2011; Sharma and Kramer, 2013), as implemented in MORPHEUS (Minguet et al., 2015). All binding site sequences identified above the threshold (>5, Supplementary Figure S2) are listed in Supplementary Table S4. Three putative AP3 binding sites were identified for *ThtAP3-1*, located at positions (upstream from start codon) -262, -139, and -121 (**Figure 5B**). *ThtAP3-2a* had only one binding site (position -9; **Figure 5C**), *ThtAP3-2b* had three (positions -293, -151, and -78; **Figure 5D**), and *ThtPI* had one (position -340; **Figure 5E**). Binding sites at position -121 in the ThtAP3-1 promoter, -91 in the ThtAP3-2a promoter, and -78 in the ThtAP3-2b promoter appear to be homologous based on our alignments with additional ranunculids (Supplementary Figure S3), and are the most conserved. They are 86.7% identical between ThtAP3-1 and ThtAP3-2b, 66.7% between ThtAP3-1 and ThtAP3-2a, and 80% between ThtAP3-2a and ThtAP3-2b (purple boxes in **Figure 5F**). The other binding sites are locus-specific (**Figure 5F**). The alignment to orthologs from other Ranunculaceae also shows the conservation of these binding sites within the family, and the sequence divergence between *ThAP3-2a* and *RanAP32-b* (Supplementary Figure S3). The *AP3-2* alignment reveals that two of *ThtAP3-2b* motifs have corresponding homologous sequence in *ThtAP3-2a* (Supplementary Figure S3B), yet they have diverged enough that their MORPHEUS scores (-1 and -3.1) were below the set threshold (**Figure 5F**, shown in lighter shading), suggesting that they are no longer functional.

Protein structural analysis on amino-acid translations of the coding sequences of all three AP3 loci identified the two alpha helices spanning the I and K domains (Supplementary Figure S4). These helices have been shown to be critical for SEP3 multimerization, and possibly for MADS box protein interactions more generally (Puranik et al., 2014). Despite its C-terminus truncation, *ThtAP3-2a* appears to possess the required helices for dimer and tetramer formation. The first helix, involved in dimer formation, has six amino acid disparities between the two paralogs; the second helix, involved in tetramer formation (Puranik et al., 2014), has 12 amino acid disparities. Of these, five involve hydrophobic residues in one paralog or the other, and a broader alignment across multiple ranunculid taxa revealed that three of these are specific to AP3-2a protein orthologs (Supplementary Figure S4). Further functional testing, combined with protein crystallography, would be necessary to ascertain potential effects of divergent residues in mediating dimerization or tetramerization between ThtAP3-2a and other MADS-box proteins.

## Discussion

This study investigated the function of three *AP3* orthologs representing ancient as well as recent gene duplications in *T. thalictroides*, a representative of the sister lineage to core eudicots. We enquired whether these gene paralogs have remained redundant or diverged in function, and whether they contribute to ectopic petaloidy of sepals. Down-regulation of two of the paralogs (*ThtAP3-1* and *ThtAP3-2b*) correlated with sepal, stamen, and stigma developmental defects. In addition to the expected interaction with the E-class protein ThtSEP3 and the other member of the B-class lineage ThtPI, all ThtAP3s had the ability to form homodimers and to interact with each other, providing a potential mechanism for novel function. Putative binding motifs were identified in all B-class gene promoters, supporting the potential for cross-regulatory interactions. Based on the evidence presented here, we conclude that, in addition to a conserved role in stamen identity, *ThtAP3-1* and *ThtAP3-2b* contribute to ectopic petaloidy of sepals in this species, as evidenced by their effect on sepal color, shape, and size. They also appear to regulate the genetic pathway leading to stigmatic papillae in carpels. Finally, we propose two working hypotheses to explain the unexpected up-regulation of all B-class genes in plants targeted for silencing of *ThtAP3-2a*, which codes for a truncated protein.

Prior characterization of *Thalictrum AP3* expression found high expression of all three loci in sepals and stamens of *T. thalictroides*, yet little to no expression in the reduced green sepals of *T. dioicum* (Di Stilio et al., 2005; LaRue et al., 2013). Together with the phenotypes emerging from our VIGS experiments, these data suggest a combined function of *ThtAP3* genes in stamen identity and in the partial transference of petaloid features to perianth organs, otherwise described as sepals based on morphology and evolutionary context (e.g., they have multiple vascular strands, they completely enclose the floral bud throughout development, and petaloid sepals are present throughout the Ranunculaceae). Even though there is no prior expression data for stigmas, our VIGS data suggest that *ThAP3* influence the development of stigmatic papillae. The conical/papillate-cell identity gene *MIXTA* is under the control of B-class genes in *Antirrhinum* and *Phalaenopsis* (Perez-Rodriguez et al., 2005; Manchado-Rojo et al., 2012; Pan et al., 2014) and is expressed in stigmatic papillae of *Thalictrum* (Di Stilio et al., 2009). The loss of stigmatic papillae in carpels of flowers experiencing down-regulation of *ThtAP3-1* and *ThtAP3-2b* in our VIGS experiments suggests that B-class regulation of *MIXTA* possibly also occurs in *Thalictrum*.

Petaloid appearance can have different genetic underpinnings, with certain species relying on B-class gene expression for ectopic petaloidy (e.g., tulips; van Tunen et al., 1993; Kanno et al., 2003), while others exhibit petaloid morphology independent of B-class gene expression (e.g., *Aristolochia*; Pabón-Mora et al., 2015). E-class genes are also known to contribute to petaloid features of sepals in *Thalictrum* (Soza et al., 2016). Given the presence of petaloid sepals throughout Ranunculaceae, it is unlikely that they arose *de novo* in *Thalictrum*. However, *Thalictrum AP3s* have a distinct role in petaloid morphology of sepals when compared to other ranunculids where it has been characterized: *Aquilegia* (Sharma and Kramer, 2013) and *Nigella* (Wang et al., 2015) in the Ranunculaceae and *Papaver* (Drea et al., 2007) in the Papaveraceae. For example, in *Aquilegia*, loss of *AqPI* function results in loss of anthocyanin production in sepals, but does not alter other aspects of sepal morphology (Sharma and Kramer, 2017). Green streaks on sepals in our VIGS experiments are comparable to those found in sepals of *Nigella damascena* loss-of-B-function mutants, which also lost anthocyanin pigmentation. In spite of this, B genes were not interpreted as playing a role in sepal petaloidy in that system (Wang et al., 2015). In *Thalictrum*, we interpreted a change toward a more leaf-like appearance of perianth organs, such as change in shape, reduction in size and the gain of photosynthetic pigment, as loss of “petaloid” features. The fact that AP3 and PI negatively regulate genes involved in chlorophyll accumulations resulting in the white *A. thaliana* petals (Mara et al., 2010), is consistent with our observations of sepal greening during down-regulation of B-class genes in *Thalictrum*, and points to similar mechanisms for this petaloid feature in petals and sepals. Based on evidence presented here, we propose that the common ancestor of *Thalictrum* evolved novel interactions among B-class proteins following its lineage-specific *ThtAP3-2a/b* gene duplication. This, in turn, enabled new regulatory interactions that contributed to sepal petaloidy in conjunction with the E-class partners (Soza et al., 2016). Consistent with this hypothesis, the wind-pollinated species *T. dioicum* has small, green sepals that do not express the *AP3-2* loci early in development (Di Stilio et al., 2005).

Given that orthologs of the A-class gene *APETALA1* do not exist in the order Ranunculales (Litt and Irish, 2003), it is likely that the genetic factors involved in *Thalictrum* sepal identity differ from those in the evolutionarily derived core eudicots. For example, the presence of chimeric outer stamens with combined carpel and sepal features in TRV2-*ThtAP3-1* and TRV2-*ThtAP3-2b* treated flowers could be considered inconsistent with the mutually antagonistic nature of the A- (sepal identity) and C- (carpel identity) classes (Bowman et al., 1991), as defined in the original ABC model. Alternatively, the presence of ectopic carpeloid features may suggest that, like in *Arabidopsis*, *T. thalictroides* B-class genes act as suppressors of carpel development genes outside of the carpel zone (Wuest et al., 2012). In *Nigella*, *AP3* homologs function in keeping the stamen–petal boundary (Gonçalves et al., 2013; Wang et al., 2015). The presence of sepal/stamen intermediates in the boundary region between these two organs in *Thalictrum* knockdowns suggests that, in the absence of petals and of the *AP3-3* petal identity gene, the other *AP3* paralogs perform a boundary-keeping role between the stamen and perianth zones (sepals, in this case). This observation is consistent with a role of B-class genes in regulating the expression zone of the C-class gene *AG* in other ranunculids (Lange et al., 2013; Sharma and Kramer, 2017). No carpel–sepal chimeras were observed in plants targeted for *ThtAP3-2a* silencing, which were actually over-expressing B-class genes. TRV2-*ThtAP3-2a* treated flowers also never exhibited signs of loss of petaloidy, such as smaller, narrower, or green sepals, and they were the only treatment to cause stunted and unfused carpels, a potential sign of B function disrupting normal carpel identity in the inner floral zone. Presence of other phenotypes, such as stamen–sepal chimeras and lobed sepals, may be explained by an overall disruption of the protein ratios necessary for normal development.

Down-regulation of the *ThtAP3* paralogs individually did not result in complete homeotic conversion of stamens into carpels or complete loss of petaloidy, suggesting at least a partial degree of redundancy. This is unlike the single copy *PI* ortholog *ThtPI*, which results in complete loss of stamen identity and a full conversion to carpels upon down-regulation (LaRue et al., 2013). Loss of petaloid features was also partial in *ThtPI* knockdowns, consisting of green streaks on otherwise white sepals, suggesting partial redundancy of this function with other B-class genes, and likely also E-class genes (LaRue et al., 2013; Soza et al., 2016). Incomplete homeotic conversion of floral organs can be attributed to partial redundancy among the *ThAP3* paralogs, supported by the presence of similar phenotypes in different treatments. This scenario differs from *Aquilegia*, in which the three *AqAP3* paralogs have sub-functionalized to stamen or petal identity (petals are absent in *Thalictrum* and so is the petal-identity paralog *AP3-3*) and neo-functionalized to staminodia identity (a fifth type of organ not present in *Thalictrum*) (Sharma et al., 2011; Sharma and Kramer, 2013). Additional experiments using double and triple constructs to target multiple genes will be needed to fully dissect the degree of redundancy amongst the *Thalictrum AP3* paralogs.

**FIGURE 6:**
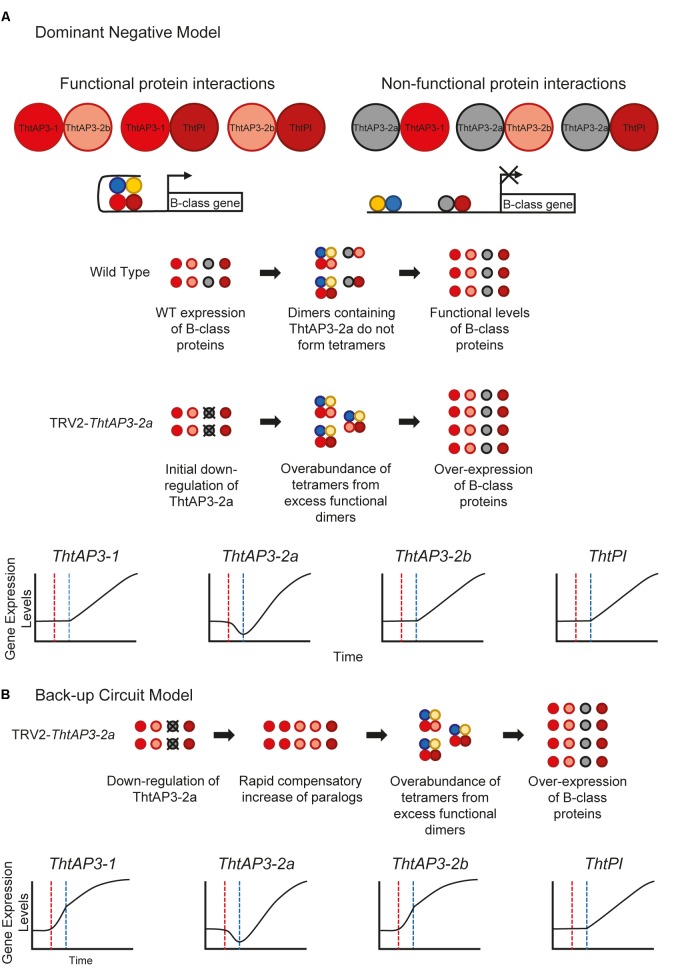
Hypothetical models for B-class gene regulation in *Thalictrum thalictroides.* **(A)** Dominant Negative Model. ThtAP3-2a is presumably able to form protein dimers (K domain intact), either hetero-dimers (shown) or homo-dimers (not shown). These “non-functional” dimers are unable to form the tetramers needed to drive transcription, as required for positive auto- and cross-regulation. ThtAP3-1 and ThtAP3-2b form protein complexes that positively auto- and cross-regulate, driving transcription of the other B-class genes. After *ThtAP3-2a* is down-regulated, as a result of targeted gene silencing (red line in graphs), the higher ratio of functional to non-functional dimers creates an overabundance of tetramers, which drives the over-expression of all B-class genes (blue line). **(B)** Back-up Circuit Model. In response to the initial down-regulation of *ThtAP3-2a* (dotted red line in graphs) during targeted gene silencing, expression of *ThtAP3-1* and *ThtAP3-2b* is up-regulated by a (as yet unknown) compensatory mechanism. Higher expression of *ThtAP3-1* and *ThtAP3-2b* initiates an excess of positive auto and cross-regulation due to total protein levels, as opposed to protein ratios in the Dominant Negative Model (note different shapes of curves from panel **A**), causing an increase in the expression of all B-class genes (dotted blue line).

The up-regulation of all B-class genes in plants targeted for *ThtAP3-2a* silencing was unexpected and, barring a general issue with our VIGS experiments, we propose two (admittedly speculative) working hypotheses leading to testable predictions for future experiments. The truncated ThtAP3-2a protein could be acting as a dominant negative (**Figure 6A**), or there could be a unidirectional back-up circuit effect (**Figure 6B**). Under the dominant negative hypothesis, ThtAP3-2a is able to form protein dimers (**Figure 4A**), but unable to form tetramers. A dominant negative regulation may arise from mutations in ThtAP3-2a affecting hydrophobic residues in the K helices (Supplementary Figure S4) that are key to tetramerization (Puranik et al., 2014), or from the loss of the highly conserved C-terminal motifs (the paleo-AP3 region and PI motif-derived; Kramer et al., 1998). While domain-swap and rescue experiments in *Arabidopsis*, including a truncated *Chloranthus* AP3, have demonstrated that the C domain and its motifs do not affect protein function (Piwarzyk et al., 2007; Su et al., 2008), the ability for the ranunculid *Eschscholzia californica* PI ortholog SEIRENA to form tetramers is dependent on five conserved residues within the PI motif (Lange et al., 2013). One future direction to test whether ThtAP3-2a is acting as a dominant negative would be to overexpress it, with the prediction that it would result in a loss of B-function phenotype; unfortunately stable transformation protocols are currently not available for *Thalictrum*. Alternatively, back-up circuits have been proposed as a mechanism in paralog retention, where one paralog compensates for the decreased expression of a partner gene (**Figure 4B**; Kafri et al., 2005, 2006). The variable distribution of AP3 binding sites among the three *ThtAP3* promoters (**Figure 5F**) lends support to this hypothesis, as paralogs with partially overlapping regulatory motifs are more efficient at rescuing the function of their mutated counterpart than paralogs with highly similar or highly dissimilar sets of motifs (Kafri et al., 2005). Likewise, the presence of more AP3 binding sites in the promoters of *ThtAP3-1* and *ThtAP3-2b* supports the hypothesis that these genes are “backing-up” *ThtAP3-2a*. Both hypothetical models rely on an initial *ThtAP3-2a* down-regulation to trigger an increase in the positive regulation of all B-class genes. The back-up model presumably leads to a more immediate increase in expression of *ThtAP3-1* and *ThtAP3-2b*, as there is also a primary “back-up” response of these paralogs (by an unknown mechanism, note different shape of gene expression curves in **Figure 6A** versus **Figure 6B**). Since we were unable to detect the putative transient down-regulation of *ThtAP3-2a*, a more comprehensive expression analysis would need to be done during earlier developmental stages to provide evidence for either of these arguments. Additional experiments are also needed to test identified binding sites *in silico*, and to ascertain whether divergent amino acids or lack of C-terminal motifs negatively affect ThtAP3-2a function.

As a representative of an early diverging eudicot, *Thalictrum* is in a key phylogenetic position to study the evolution of floral MADS box protein–protein interactions. Our yeast two-hybrid assays showed novel and promiscuous interactions among the different *Thalictrum* AP3s. Core eudicot EuAP3 and most monocot paleoAP3 proteins do not homodimerize, but rather interact as obligate heterodimers with PI (Schwarz-Sommer et al., 1992; Tröbner et al., 1992; Riechmann et al., 1996; Moon et al., 1999; Vandenbussche et al., 2004), and this may have led to the canalization and increased robustness of the eudicot flower (Lenser et al., 2009; Melzer et al., 2014). However, AP3 and PI orthologs from early-diverging angiosperms and monocots are able to interact both as homo- and heterodimers (Melzer et al., 2014). AP3 homodimerization was probably lost very early in angiosperm evolution (before the eudicot–monocot split), while PI homodimerization was likely lost later, but before the diversification of the eudicots (Melzer et al., 2014). Evidence presented here suggests that *Thalictrum* floral MADS-box protein biochemical behavior more closely matches that of early diverging angiosperms than that of core eudicots. *Aquilegia vulgaris* AP3 (AqvAP3-2 and AqvAP3-3) have also been shown to homodimerize, yet the three proteins do not dimerize with each other (Kramer et al., 2007). All three *Thalictrum* AP3s not only heterodimerize with ThtPI as expected, but also homodimerize and heterodimerize with each other (**Figure 4**). In addition, all four B-class protein products can interact with the E-class partner ThtSEP3. While certain interactions only occurred in one direction, similar asymmetrical results have been observed, and deemed valid, in previous publications for other MADS box genes (e.g., Galimba et al., 2012; Lange et al., 2013). Yeast two-hybrid experiments provide evidence for biochemical interaction amongst the candidate proteins and are typically used as proxy; further studies will be needed to confirm whether these interactions occur *in planta.* Taken together, our results suggest that ThtAP3s can form homodimers, in addition to the AP3-PI heterodimers that preferentially populate B-class/E-class tetramers in *Arabidopsis* (Melzer and Theißen, 2009). Our detection of AP3 binding sites in the promoters of all B-class genes (**Figure 5**) supports auto- and cross-gene regulation, as has been described for MADS box genes more generally (Kaufmann et al., 2009). We therefore propose that these novel protein interactions provide a potential mechanism for the role of *ThtAP3s* in ectopic petaloidy of sepals.

Although gene duplication has long been recognized as an important contributor to the evolution of biological complexity (Ohno, 1970; Force et al., 1999), functional studies showing the fate of duplicated developmental genes are still limited. Here, we analyzed the function of duplicated *AP3* orthologs from a basal eudicot and found deep conservation in stamen identity, a novel role in ectopic petaloidy and stigma development, and partial functional redundancy. While additional experiments will be needed to fully dissect the complex genetic interactions uncovered by our work, evidence presented here lends further support to the overarching hypothesis that the duplication of floral organ identity genes contributed to angiosperm diversification via the generation of floral diversity.

## Author Contributions

KG performed the experiments and wrote the manuscript. JM-G performed the yeast two-hybrid and promoter analysis and assisted with methods, descriptions, and supplementary materials. VDS designed the study, secured the funding, coordinated the experiments, and edited the manuscript.

## Conflict of Interest Statement

The authors declare that the research was conducted in the absence of any commercial or financial relationships that could be construed as a potential conflict of interest.

## Footnotes

1 https://sites.google.com/a/ualberta.ca/onekp

2 http://jaspar.genereg.net

3 http://www.rsat.eu

4 http://biodev.cea.fr/morpheus/

## References

[B1] Airoldi C. A. Davies B. (2012). Gene duplication and the evolution of plant MADS-box transcription factors. *J. Genet. Genomics* 39 157–165. 10.1016/j.jgg.2012.02.008 22546537

[B2] Arango-Ocampo C. González F. Alzate J. F. Pabón-Mora N. (2016). The developmental and genetic bases of apetaly in *Bocconia frutescens* (Chelidonieae: Papaveraceae). *EvoDevo* 7:16. 10.1186/s13227-016-0054-6 27489612 PMC4971710

[B3] Bowman J. L. Smyth D. R. Meyerowitz E. M. (1989). Genes directing flower development in Arabidopsis. *Plant Cell* 1 37–52. 10.1105/tpc.1.1.37 2535466 PMC159735

[B4] Bowman J. L. Smyth D. R. Meyerowitz E. M. (1991). Genetic interactions among floral homeotic genes of Arabidopsis. *Development* 112 1–20. 1685111 10.1242/dev.112.1.1

[B5] Causier B. Davies B. (2002). Analysing protein-protein interactions with the yeast two-hybrid system. *Plant Mol. Biol.* 50 855–870. 10.1023/A:1021214007897 12516858

[B6] Coen E. S. Meyerowitz E. M. (1991). The war of the whorls: genetic interactions controlling flower development. *Nature* 353 31–37. 10.1038/353031a0 1715520

[B7] Cronk Q. C. B. Bateman R. M. Hawkins J. A. (2002). *Developmental Genetics and Plant Evolution.* Boca Raton, FL: CRC Press.

[B8] Davies B. Egea-Cortines M. de Andrade Silva E. Saedler H. Sommer H. (1996). Multiple interactions amongst floral homeotic MADS box proteins. *EMBO J.* 15 4330–4343. 8861961 PMC452158

[B9] Di Stilio V. S. (2011). Empowering plant evo-devo: virus induced gene silencing validates new and emerging model systems. *Bioessays* 33 711–718. 10.1002/bies.201100040 21744374

[B10] Di Stilio V. S. Kramer E. M. Baum D. A. (2005). Floral MADS box genes and homeotic gender dimorphism in *Thalictrum dioicum* (Ranunculaceae) - a new model for the study of dioecy. *Plant J.* 41 755–766. 10.1111/j.1365-313X.2005.02336.x 15703062

[B11] Di Stilio V. S. Kumar R. A. Oddone A. M. Tolkin T. R. Salles P. McCarty K. (2010). Virus-induced gene silencing as a tool for comparative functional studies in *Thalictrum*. *PLoS One* 5:e12064. 10.1371/journal.pone.0012064 20706585 PMC2919395

[B12] Di Stilio V. S. Martin C. Schulfer A. F. Connelly C. F. (2009). An ortholog of MIXTA-like2 controls epidermal cell shape in flowers of *Thalictrum*. *New Phytol.* 183 718–728. 10.1111/j.1469-8137.2009.02945.x 19659588

[B13] Drea S. Hileman L. C. de Martino G. Irish V. F. (2007). Functional analyses of genetic pathways controlling petal specification in poppy. *Development* 134 4157–4166. 10.1242/dev.013136 17959716

[B14] Edgar R. C. (2004). MUSCLE: multiple sequence alignment with high accuracy and high throughput. *Nucleic Acids Res.* 32 1792–1797. 10.1093/nar/gkh340 15034147 PMC390337

[B15] Force A. Lynch M. Pickett F. B. Amores A. Yan Y. Postlethwait J. (1999). Preservation of duplicate genes by complementary, degenerative mutations. *Genetics* 151 1531–1545. 10101175 10.1093/genetics/151.4.1531 PMC1460548

[B16] Galimba K. D. (2015). *Duplication and Functional Divergence in the Floral Organ Identity Genes.* Ph.D. thesis, University of Washington, Seattle, WA.

[B17] Galimba K. D. Tolkin T. R. Sullivan A. M. Melzer R. Theißen G. Di Stilio V. S. (2012). Loss of deeply conserved C-class floral homeotic gene function and C- and E-class protein interaction in a double-flowered ranunculid mutant. *Proc. Natl. Acad. Sci. U.S.A.* 109 E2267–E2275. 10.1073/pnas.1203686109 22853954 PMC3427126

[B18] Gonçalves B. Nougué O. Jabbour F. Ridel C. Morin H. Laufs P. (2013). An APETALA3 homolog controls both petal identity and floral meristem patterning in *Nigella damascena* L. (Ranunculaceae). *Plant J.* 76 223–235. 10.1111/tpj.12284 23855996

[B19] Heard J. Dunn K. (1995). Symbiotic induction of a MADS-box gene during development of alfalfa root nodules. *Proc. Natl. Acad. Sci. U.S.A.* 92 5273–5277. 10.1073/pnas.92.12.5273 7777496 PMC41676

[B20] Immink R. G. Tonaco I. A. de Folter S. Shchennikova A. van Dijk A. D. Busscher-Lange J. (2009). SEPALLATA3: the “glue” for MADS box transcription factor complex formation. *Genome Biol.* 10:R24. 10.1186/gb-2009-10-2-r24 19243611 PMC2688274

[B21] Jaramillo M. A. Kramer E. M. (2004). *APETALA3* and *PISTILLATA* homologs exhibit novel expression patterns in the unique perianth of Aristolochia (Aristolochiaceae). *Evol. Dev.* 6 449–458. 10.1111/j.1525-142X.2004.04053.x 15509227

[B22] Jaramillo M. A. Kramer E. M. (2007). Molecular evolution of the petal and stamen identity genes, *APETALA3* and *PISTILLATA*, after petal loss in the Piperales. *Mol. Phylogenet. Evol.* 44 598–609. 10.1016/j.ympev.2007.03.015 17576077

[B23] Kafri R. Bar-Even A. Pilpel Y. (2005). Transcription control reprogramming in genetic backup circuits. *Nat. Genet.* 37 295–299. 10.1038/ng1523 15723064

[B24] Kafri R. Levy M. Pilpel Y. (2006). The regulatory utilization of genetic redundancy through responsive backup circuits. *Proc. Natl. Acad. Sci. U.S.A.* 103 11653–11658. 10.1073/pnas.0604883103 16861297 PMC1513536

[B25] Kanno A. Saeki H. Kameya T. Saedler H. Theissen G. (2003). Heterotopic expression of class B floral homeotic genes supports a modified ABC model for tulip (*Tulipa gesneriana*). *Plant Mol. Biol.* 52 831–841. 10.1023/A:1025070827979 13677470

[B26] Kaufmann K. Muiño J. M. Jauregui R. Airoldi C. A. Smaczniak C. Krajewski P. (2009). Target genes of the MADS transcription factor SEPALLATA3: integration of developmental and hormonal pathways in the *Arabidopsis* flower. *PLoS Biol.* 7:e1000090. 10.1371/journal.pbio.1000090 19385720 PMC2671559

[B27] Kelley L. A. Mezulis S. Yates C. M. Wass M. N. Sternberg M. J. E. (2015). The Phyre2 web portal for protein modeling, prediction and analysis. *Nat. Protoc.* 10 845–858. 10.1038/nprot.2015.053 25950237 PMC5298202

[B28] Kim S. Yoo M.-J. Albert V. A. Farris J. S. Soltis P. S. Soltis D. E. (2004). Phylogeny and diversification of B-function MADS-box genes in angiosperms: evolutionary and functional implications of a 260-million-year-old duplication. *Am. J. Bot.* 91 2102–2118. 10.3732/ajb.91.12.2102 21652358

[B29] Kramer E. M. Di Stilio V. S. Schluter P. (2003). Complex patterns of gene duplication in the *APETALA3* and *PISTILLATA* lineages of the Ranunculaceae. *Int. J. Plant Sci.* 164 1–11. 10.1086/344694

[B30] Kramer E. M. Dorit R. L. Irish V. F. (1998). Molecular evolution of genes controlling petal and stamen development: duplication and divergence within the APETALA3 and PISTILLATA MADS-box gene lineages. *Genetics* 149 765–783. 9611190 10.1093/genetics/149.2.765 PMC1460198

[B31] Kramer E. M. Holappa L. Gould B. Jaramillo M. A. Setnikov D. Santiago P. M. (2007). Elaboration of B gene function to include the identity of novel floral organs in the lower eudicot *Aquilegia*. *Plant Cell* 19 750–766. 10.1105/tpc.107.050385 17400892 PMC1867376

[B32] Krizek B. A. Meyerowitz E. M. (1996). The Arabidopsis homeotic genes APETALA3 and PISTILLATA are sufficient to provide the B class organ identity function. *Development* 122 11–22. 8565821 10.1242/dev.122.1.11

[B33] Lange M. Orashakova S. Lange S. Melzer R. Theißen G. Smyth D. R. (2013). The seirena B class floral homeotic mutant of California poppy (*Eschscholzia californica*) reveals a function of the enigmatic PI motif in the formation of specific multimeric MADS domain protein complexes. *Plant Cell* 25 438–453. 10.1105/tpc.112.105809 23444328 PMC3608770

[B34] LaRue N. C. Sullivan A. M. Stilio V. S. D. (2013). Functional recapitulation of transitions in sexual systems by homeosis during the evolution of dioecy in *Thalictrum*. *Front. Plant Sci.* 4:487. 10.3389/fpls.2013.00487 24348491 PMC3842162

[B35] Lenser T. Theißen G. Dittrich P. (2009). Developmental robustness by obligate interaction of class B floral homeotic genes and proteins. *PLoS Comput. Biol.* 5:e1000264. 10.1371/journal.pcbi.1000264 19148269 PMC2612583

[B36] Litt A. Irish V. F. (2003). Duplication and diversification in the APETALA1/FRUITFULL floral homeotic gene lineage: implications for the evolution of floral development. *Genetics* 165 821–833. 14573491 10.1093/genetics/165.2.821 PMC1462802

[B37] Litt A. Kramer E. M. (2010). The ABC model and the diversification of floral organ identity. *Semin. Cell Dev. Biol.* 21 129–137. 10.1016/j.semcdb.2009.11.019 19948236

[B38] Liu Y. L. Schiff M. Dinesh-Kumar S. P. (2002). Virus-induced gene silencing in tomato. *Plant J.* 31 777–786. 10.1046/j.1365-313X.2002.01394.x 12220268

[B39] Livak K. J. Schmittgen T. D. (2001). Analysis of relative gene expression data using real-time quantitative PCR and the 2^-ΔΔ*Ct*^ method. *Methods* 25 402–408. 10.1006/meth.2001.1262 11846609

[B40] Maia V. H. Gitzendanner M. A. Soltis P. S. Wong G. K.-S. Gane Ka-Shu Soltis D. E. (2014). Angiosperm phylogeny based on 18S/26S rDNA sequence data: constructing a large data set using next-generation sequence data. *Int. J. Plant Sci.* 175 613–650. 10.1086/676675

[B41] Manchado-Rojo M. Delgado-Benarroch L. Roca M. J. Weiss J. Egea-Cortines M. (2012). Quantitative levels of *Deficiens* and *Globosa* during late petal development show a complex transcriptional network topology of B function. *Plant J.* 72 294–307. 10.1111/j.1365-313X.2012.05080.x 22708513

[B42] Mara C. D. Huang T. B. Irish V. F. (2010). The *Arabidopsis* floral homeotic proteins APETALA3 and PISTILLATA negatively regulate the BANQUO genes implicated in light signaling. *Plant Cell* 22 690–702. 10.1105/tpc.109.065946 20305124 PMC2861465

[B43] Mathelier A. Zhao X. Zhang A. W. Parcy F. Worsley-Hunt R. Arenillas D. J. (2014). JASPAR 2014: an extensively expanded and updated open-access database of transcription factor binding profiles. *Nucleic Acids Res.* 42 D142–D147. 10.1093/nar/gkt997 24194598 PMC3965086

[B44] Medina-Rivera A. Defrance M. Sand O. Herrmann C. Castro-Mondragon J. A. Delerce J. (2015). RSAT 2015: regulatory sequence analysis tools. *Nucleic Acids Res.* 43 W50–W56. 10.1093/nar/gkv362 25904632 PMC4489296

[B45] Melzer R. Härter A. Rümpler F. Kim S. Soltis P. S. Soltis D. E. (2014). DEF- and GLO-like proteins may have lost most of their interaction partners during angiosperm evolution. *Ann. Bot.* 114 1431–1443. 10.1093/aob/mcu094 24902716 PMC4204782

[B46] Melzer R. Theißen G. (2009). Reconstitution of ‘floral quartets’ *in vitro* involving class B and class E floral homeotic proteins. *Nucleic Acids Res.* 37 2723–2736. 10.1093/nar/gkp129 19276203 PMC2677882

[B47] Minguet E. G. Segard S. Charavay C. Parcy F. (2015). MORPHEUS, a webtool for transcription factor binding analysis using position weight matrices with dependency. *PLoS One* 10:e0135586. 10.1371/journal.pone.0135586 26285209 PMC4540572

[B48] Moon Y. H. Jung J. Y. Kang H. G. An G. (1999). Identification of a rice APETALA3 homologue by yeast two-hybrid screening. *Plant Mol. Biol.* 40 167–177. 10.1023/A:1026429922616 10394955

[B49] Ohno S. (1970). *Evolution by Gene Duplication.* Berlin: Springer-Verlag 10.1007/978-3-642-86659-3

[B50] Pabón-Mora N. Suárez-Baron H. Ambrose B. A. González F. (2015). Flower development and perianth identity candidate genes in the basal angiosperm *Aristolochia fimbriata* (Piperales: Aristolochiaceae). *Front. Plant Sci.* 6:1095. 10.3389/fpls.2015.01095 26697047 PMC4675851

[B51] Pan Z.-J. Chen Y.-Y. Du J.-S. Chen Y.-Y. Chung M.-C. Tsai W.-C. (2014). Flower development of *Phalaenopsis* orchid involves functionally divergent SEPALLATA-like genes. *New Phytol.* 202 1024–1042. 10.1111/nph.12723 24571782 PMC4288972

[B52] Pelaz S. Gustafson-Brown C. Kohalmi S. E. Crosby W. L. Yanofsky M. F. (2001). APETALA1 and SEPALLATA3 interact to promote flower development. *Plant J.* 26 385–394. 10.1046/j.1365-313X.2001.2641042.x 11439126

[B53] Perez-Rodriguez M. Jaffe F. W. Butelli E. Glover B. J. Martin C. (2005). Development of three different cell types is associated with the activity of a specific MYB transcription factor in the ventral petal of *Antirrhinum majus* flowers. *Development* 132 359–370. 10.1242/dev.01584 15604096

[B54] Piwarzyk E. Yang Y. Jack T. (2007). Conserved C-terminal motifs of the Arabidopsis proteins APETALA3 and PISTILLATA are dispensable for floral organ identity function. *Plant Physiol.* 145 1495–1505. 10.1104/pp.107.105346 17965182 PMC2151695

[B55] Puranik S. Acajjaoui S. Conn S. Costa L. Conn V. Vial A. (2014). Structural basis for the oligomerization of the MADS domain transcription factor SEPALLATA3 in *Arabidopsis*. *Plant Cell* 26 3603–3615. 10.1105/tpc.114.127910 25228343 PMC4213154

[B56] Riechmann J. L. Krizek B. A. Meyerowitz E. M. (1996). Dimerization specificity of Arabidopsis MADS domain homeotic proteins APETALA1 APETALA3 PISTILLATA, and AGAMOUS. *Proc. Natl. Acad. Sci. U.S.A.* 93 4793–4798. 10.1073/pnas.93.10.4793 8643482 PMC39358

[B57] Schwarz-Sommer Z. Hue I. Huijser P. Flor P. J. Hansen R. Tetens F. (1992). Characterization of the Antirrhinum floral homeotic MADS-box gene deficiens: evidence for DNA binding and autoregulation of its persistent expression throughout flower development. *EMBO J.* 11 251–263. 1346760 10.1002/j.1460-2075.1992.tb05048.x PMC556446

[B58] Sharma B. Guo C. Kong H. Kramer E. M. (2011). Petal-specific subfunctionalization of an APETALA3 paralog in the Ranunculales and its implications for petal evolution. *New Phytol.* 191 870–883. 10.1111/j.1469-8137.2011.03744.x 21557746

[B59] Sharma B. Kramer E. (2013). Sub- and neo-functionalization of *APETALA3* paralogs have contributed to the evolution of novel floral organ identity in *Aquilegia* (columbine, Ranunculaceae). *New Phytol.* 197 949–957. 10.1111/nph.12078 23278258

[B60] Sharma B. Kramer E. M. (2017). *Aquilegia* B gene homologs promote petaloidy of the sepals and maintenance of the C domain boundary. *Evodevo* 8:22. 10.1186/s13227-017-0085-7 29209492 PMC5704387

[B61] Soltis D. E. Smith S. A. Cellinese N. Wurdack K. J. Tank D. C. Brockington S. F. (2011). Angiosperm phylogeny: 17 genes, 640 taxa. *Am. J. Bot.* 98 704–730. 10.3732/ajb.1000404 21613169

[B62] Soltis P. Soltis D. (2014). “Flower diversity and angiosperm diversification,” in *Flower Development*, eds Riechmann J. L. Wellmer F. (New York, NY: Springer), 85–102. 10.1007/978-1-4614-9408-9_4 24395253

[B63] Soukup S. W. (1974). “Evolution by gene duplication,” in *Teratology* Vol. 9 ed. Ohno S. (New York, NY: Springer-Verlag), 250–251.

[B64] Soza V. L. Brunet J. Liston A. Salles Smith P. Di Stilio V. S. (2012). Phylogenetic insights into the correlates of dioecy in meadow-rues (*Thalictrum*, Ranunculaceae). *Mol. Phylogenet. Evol.* 63 180–192. 10.1016/j.ympev.2012.01.009 22289865

[B65] Soza V. L. Snelson C. D. Hewett Hazelton K. D. Di Stilio V. S. (2016). Partial redundancy and functional specialization of E-class SEPALLATA genes in an early-diverging eudicot. *Dev. Biol.* 419 143–155. 10.1016/j.ydbio.2016.07.021 27502434

[B66] Stormo G. D. (2013). Modeling the specificity of protein-DNA interactions. *Quant. Biol.* 1 115–130. 10.1007/s40484-013-0012-4 25045190 PMC4101922

[B67] Su K. Zhao S. Shan H. Kong H. Lu W. Theissen G. (2008). The MIK region rather than the C-terminal domain of AP3-like class B floral homeotic proteins determines functional specificity in the development and evolution of petals. *New Phytol.* 178 544–558. 10.1111/j.1469-8137.2008.02382.x 18298432

[B68] Theissen G. Kim J. T. Saedler H. (1996). Classification and phylogeny of the MADS-box multigene family suggest defined roles of MADS-box gene subfamilies in the morphological evolution of eukaryotes. *J. Mol. Evol.* 43 484–516. 10.1007/BF02337521 8875863

[B69] Theißen G. Saedler H. (2001). Plant biology: floral quartets. *Nature* 409 469–471. 10.1038/35054172 11206529

[B70] Tilly J. J. Allen D. W. Jack T. (1998). The CArG boxes in the promoter of the Arabidopsis floral organ identity gene APETALA3 mediate diverse regulatory effects. *Development* 125 1647–1657. 9521903 10.1242/dev.125.9.1647

[B71] Tröbner W. Ramirez L. Motte P. Hue I. Huijser P. Lönnig W. E. (1992). GLOBOSA: a homeotic gene which interacts with DEFICIENS in the control of *Antirrhinum* floral organogenesis. *EMBO J.* 11 4693–4704. 1361166 10.1002/j.1460-2075.1992.tb05574.x PMC556944

[B72] van Tunen A. J. Eikelboom W. Angenent G. C. (1993). Floral organogenesis in *Tulipa*. *Flower. Newsl.* 16 33–37.

[B73] Vandenbussche M. Zethof J. Royaert S. Weterings K. Gerats T. (2004). The duplicated B-class heterodimer model: whorl-specific effects and complex genetic interactions in *Petunia hybrida* flower development. *Plant Cell* 16 741–754. 10.1105/tpc.019166 14973163 PMC385285

[B74] Wang P. Liao H. Zhang W. Yu X. Zhang R. Shan H. (2015). Flexibility in the structure of spiral flowers and its underlying mechanisms. *Nat. Plants* 2:15188. 10.1038/nplants.2015.188 27250746

[B75] Wuest S. E. O’Maoileidigh D. S. Rae L. Kwasniewska K. Raganelli A. Hanczaryk K. (2012). Molecular basis for the specification of floral organs by APETALA3 and PISTILLATA. *Proc. Natl. Acad. Sci. U.S.A.* 109 13452–13457. 10.1073/pnas.1207075109 22847437 PMC3421202

[B76] Zahn L. M. Leebens-Mack J. dePamphilis C. W. Ma H. Theissen G. (2005). To B or not to B a flower: the role of DEFICIENS and GLOBOSA orthologs in the evolution of the angiosperms. *J. Hered.* 96 225–240. 10.1093/jhered/esi033 15695551

[B77] Zhang R. Guo C. Zhang W. Wang P. Li L. Duan X. (2013). Disruption of the petal identity gene APETALA3-3 is highly correlated with loss of petals within the buttercup family (Ranunculaceae). *Proc. Natl. Acad. Sci. U.S.A.* 110 5074–5079. 10.1073/pnas.1219690110 23479615 PMC3612624

