# Supplementary material for: Gene Duplication and Transference of Function in the paleoAP3 Lineage of Floral Organ Identity Genes
Source: Front Plant Sci. 2018 Mar 23;9:334. doi: 10.3389/fpls.2018.00334 (PMC5876318; doi:10.3389/fpls.2018.00334)
Supplement: Supplementary file 1 [file Presentation_1.PDF]

Supplementary Material

Gene duplication and functional divergence in the paleo*AP3* lineage of floral organ identity genes

Kelsey D. Galimba, Jesús Martínez-Gómez, Verónica S. Di Stilio*

*** Correspondence:** Verónica S. Di Stilio: distilio@u.washington.edu

Supplementary Figure 1. Detection of tobacco rattle virus (TRV) transcripts in plants treated by virus-induced gene silencing. Expression analysis of plants treated with untreated controls, empty TRV2 + TRV1, TRV2-*ThtAP3-1* + TRV1, TRV2-*ThtAP3-2a* + TRV1, and TRV2-*ThtAP3-2b* + TRV1, by RT-PCR. TRV1 and TRV2 were detected only in VIGS-treated and empty TRV2-treated samples. Empty TRV2 generates a 390 bp band, while TRV2 containing *ThtAP3-1* = 819 bp, *ThtAP3-2a* = 817 bp, and *ThtAP3-2b* = 798 bp. Approximate size of bands (in bp) indicated on the right. Treatments and plant numbers are labeled above each lane.

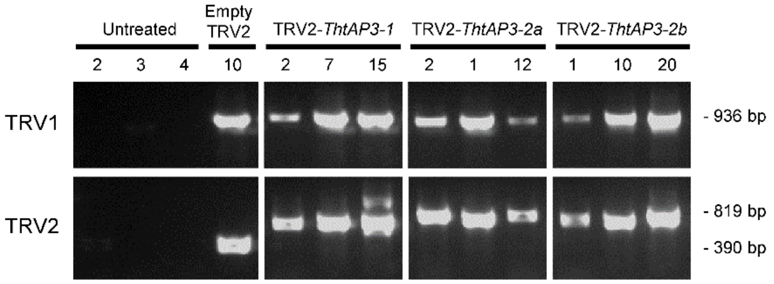

Supplementary Figure 2. MORPHEUS score distribution of transcription factor binding sites (TBS) in 1Kb fragments upstream of the start codon for the three *Thalictrum thalictroides AP3* orthologs *ThtAP3-1*, *ThtAP3-2a* and *ThtAP3-2b*, using the *Arabidopsis thaliana AP3* position-specific scoring matrix. AP3 binding sites above the threshold of 5 (dotted red line) are mapped in Fig. 5.

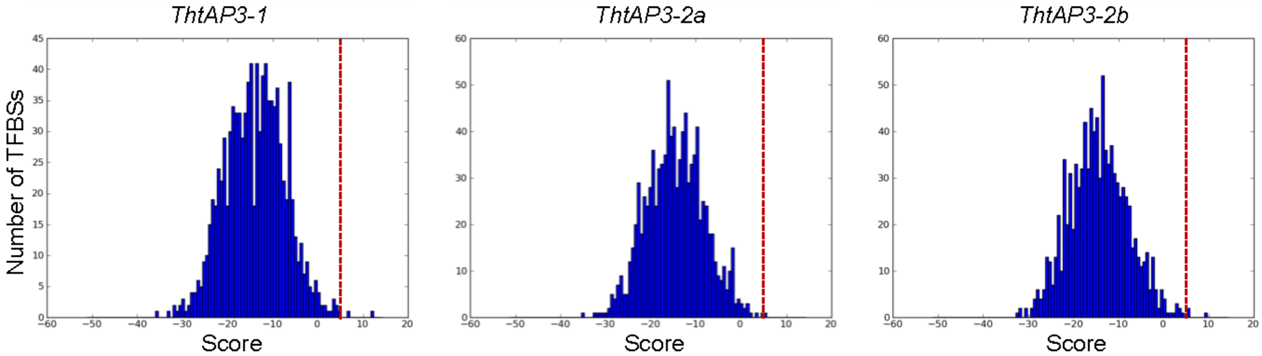

Supplementary Figure 3. Alignment of putative promoter regions for Ranunculaceae *AP3-1*, *AP3-2a* and *AP3-2b*. MUSCLE alignment of putative promoter regions of AP3 orthologs from Ranunculaceae species. A. RanAP3-1; B. RanAP3-2a; C. RanAP3-2b. Alignment consists of 500 base pairs upstream of ATG start codon, sequence number is noted on top relative to *ThtAP3* genes. Sequence logo denotes amount of conservation. Putative AP3 binding sites (containing CArG boxes) are boxed. *Tht=Thalictrum thalictroides, Thp=Thalictrum petaloideum, Lef =Leptopyrum fumarioides, Aqc=Aquilegia coerulea, Enr= Enemion raddeanum, Ism= Isopyrum manshuricum, Bec= Beesia calthifolia, Acv= Actaea vaginata, Clh= Clematis heracleifolia, Clm= Clematis macropetala, Raj= Ranunculus japonicas.*

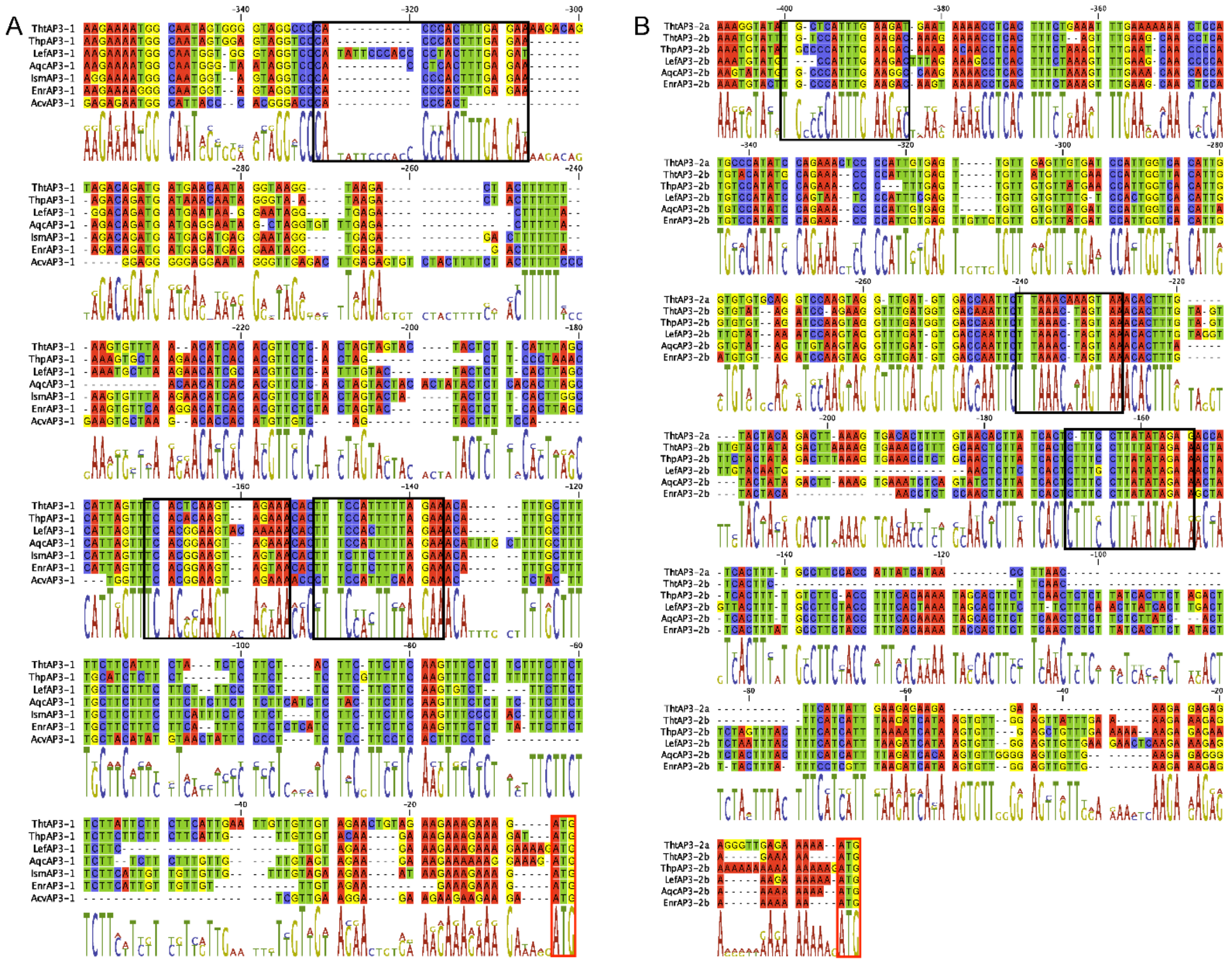

Supplementary Figure 4. Amino acid alignment of *Arabidopsis* AP3, *Thalictrum thalictroides* AP3-2a and AP3-2b and additional ranunculid AP3 orthologs showing protein domain structure and two predicted alpha-helices that span the I-, K- and C-terminus domains. MADS-domain depicted in red, I-domain in blue, K-domain in green, and C-terminus in yellow. Sequence logo illustrates conserved amino acids; filled circles denote a hydrophobic amino acid present in ThtAP3-2a and not in ThtAP3-2b, open circle denotes a hydrophobic amino acid present in ThtAP3-2b. Star indicates divergent residues unique to AP3-2a orthologs. Asterisks indicate STOP codons. Tht=*Thalictrum thalictroides*, Thd=*Thalictrum dioicum*, Ism= *Isopyrum manshuricum*, Enr= *Enemion raddeanum*, Thp=*Thalictrum petaloideum*, Aqc=*Aquilegia coerulea*, Aqv=*Aquilegia vulgaris*, Aqa=*Aquilegia alpina*, Lef =*Leptopyrum fumarioides.*

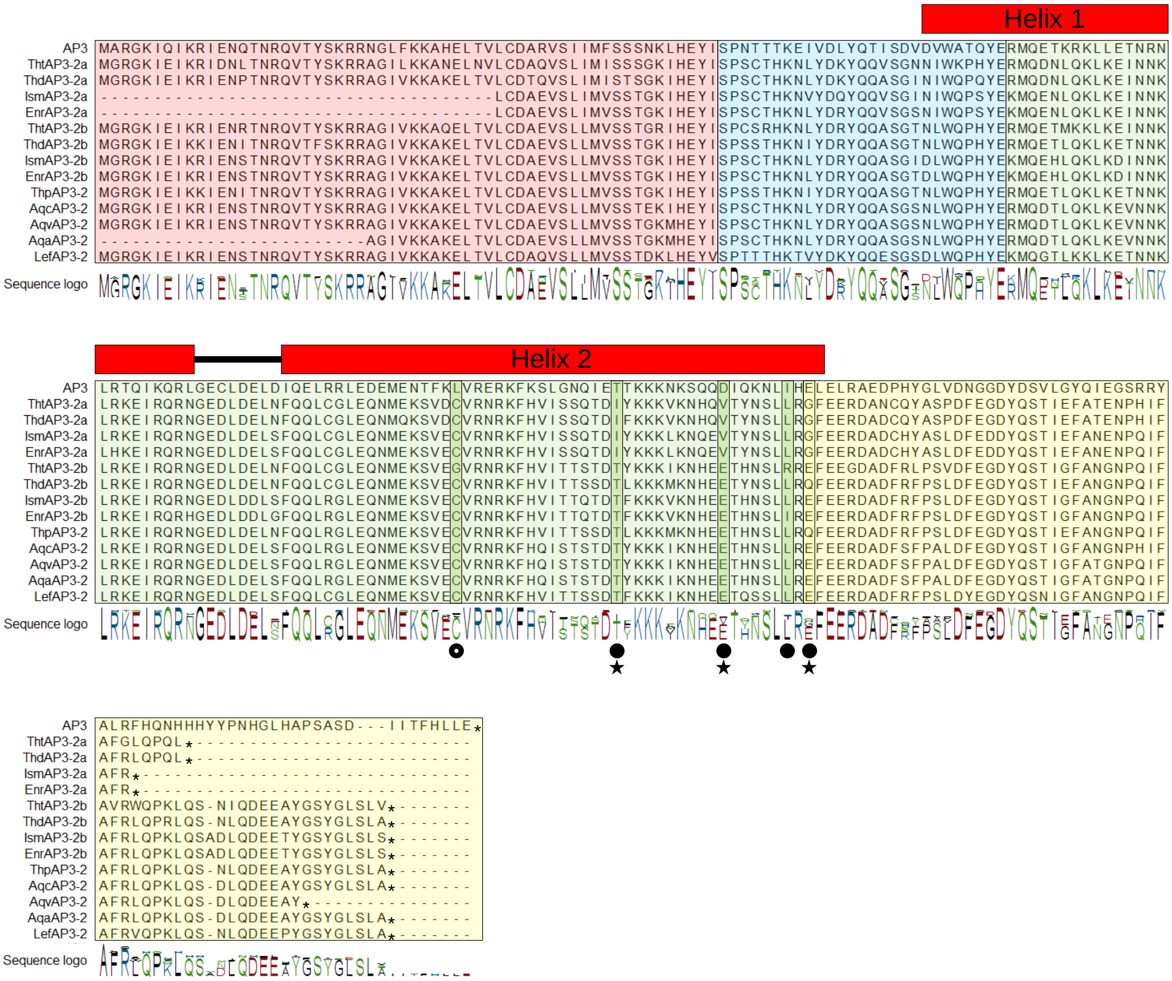

Supplementary Table 1. Sequence of primers used in this study. Blue text indicates nucleotides added for restriction digestion.

Primer Purpose	Name	Sequence
**VIGS cloning/validation**		
TthAP3-1 Construct	*TthAP3-1* F BamHI	5’ TAG GGA TCC GTG TTC GTA ATC GAA AGT TT 3’
	TthAP3-1 R KpnI	5’ GAT GGT ACC GCA GAA CAC ATA CAA GTT A 3’
TthAP3-2a Construct	*TthAP3-2a* F BamHI	5’ TAG GGA TCC GTG TTC GTA ATC GAA AGT TT 3’
	TthAP3-2a R KpnI	5’ GAT GGT ACC GCA GAA CAC ATA CAA GTT A 3’
TthAP3-2b Construct	*TthAP3-2b* F BamHI	5’ TAG GGA TCC TAC TAC TAG TAC TGA CAC TT 3’
	TthAP3-2b R KpnI	5’ GAT GGT ACC GTT CAT ACA AAA GAT CCA A 3’
Presence of TRV1	pTRV1_fwd	5’ CTT GAA GAA GAA GAC TTT CGA AGT CTC 3’
	pTRV1_rev	5’ GTA AAA TCA TTG ATA ACA ACA CAG ACA AAC 3’
Presence of TRV2	OYL195	5’ GGT CAA GGT ACG TAG TAG AG 3’
	OYL198	5’ CGA GAA TGT CAA TCT CGT AGG 3’
**Y2H cloning**	TthAP3-1_Y2H_F	5'- CAT GGA GGC CGA ATT CAT GGG GAG AGG AAA GAT TGA GAT C -3'
	TthAP3-1_Y2H_R	5'- GCA GGT CGA CGG ATC CTC AAC CTA ATC GAA GAC CCT CGA A -3'
	TthAP3-2a/b_Y2H_F	5'- CAT GGA GGC CGA ATT CAT GGG GAG AGG AAA GAT TGA GAT T -3'
	TthAP3-2a_Y2H_R	5'- GCA GGT CGA CGG ATC CTT ACA GCT GTG GTT GCA AAC CGA A -3'
	TthAP3-2b_Y2H_R	5'- GCA GGT CGA CGG ATC CTC AAA CAA GAC TTA AGC CAT ATG A -3'
	TthPI_Y2H_F	5'- CAT GGA GGC CGA ATT CAT GGG AAG AGG TAA GAT TGA GAT C -3'
	TthPI_Y2H_R	5'- GCA GGT CGA CGG ATC CCT ATT TTT CCT GTA AAT TAG GCT G -3'
**Expression by qPCR**	*ThtAP3-1*-for-qPCR	5’ GGG TTG GTG GTG AAG ATC TGA GTG ATA 3’
	ThtAP3-1-rev-qPCR	5’ ATC AAC TAG TGC ATA TGG TTC CTC AAG 3’
	ThtAP3-2a-for-qPCR	5’ GCA AGA TAA TCT GCA GAA GCT GAA GGA 3’
	ThtAP3-2a-rev-qPCR	5’ ACG TAG CAG ACC GTT ATA GGT CAC TTG 3’
	ThtAP3-2b-for-qPCR	5’ GCA AGA TAA TCT GCA GAA GCT GAA GGA 3’
	ThtAP3-2b-rev-qPCR	5’ ACG TAG CAG ACC GTT ATA GGT CAC TTG 3’
	ThtPI-for-qPCR	5’ TAC AAG TAT GCT AGA GGA AGA GAA CAA 3’
	ThtPI-rev-qPCR	5’ AGA TCT ATT CAT TAT AAG GCT CAT GGT 3’

Supplementary Table 2. List of Ranunculaceae species and accession numbers used in promoter binding site analyses of *RanAP3* loci (Fig. S3) and/or in the protein sequence alignment (Fig. S4). Asterisk indicates sequences that fall into the AP3-2b clade (Zhang et al. 2013).

Species	Locus	GenBank Accession	Reference
Actaea vaginata	AcvAP3-1	KC701410.1	Zhang et al. 2013
Aquilegia alpinum	AqcaAP3-2*	AY162850.1	Kramer et al. 2003
Aquilegia coerulea	AqcoAP3-1	N/A	Phytozome
	AqcoAP3-2b	N/A	Phytozome
Aquilegia vulgaris	AqvAP3-2*	EF489477.1	Kramer et al. 2007
Enemion raddeanum	EnrAP3-1	KC701398.1	Zhang et al. 2013
	EnrAP3-2a	KC701415.1	Zhang et al. 2013
	EnrAP3-2b	KC701399.1	Zhang et al. 2013
Isopyrum manshuricum	IsmAP3-1	KC701401.1	Zhang et al. 2013
	IsmAP3-2a	KC701416.1	Zhang et al. 2013
	IsmAP3-2b	KC701402.1	Zhang et al. 2013
Leptopyrum fumarioides	LefAP3-1	KC701404.1	Zhang et al. 2013
	LefAP3-2*	KC701405	Zhang et al. 2013
Thalictrum dioicum	ThtdAP3-2a	AY867876.1	Di Stilio et al. 2005
	ThtdAP3-2b	AY867877.1	Di Stilio et al. 2005
Thalictrum petaloideum	ThpAP3-1	KC701413	Zhang et al. 2013
	ThpAP3-2*	KC701414	Zhang et al. 2013
Thalictrum thalictroides	ThtAP3-1	MG889397	This Study
	ThtAP3-2a	MG889396	This Study
	ThtAP3-2b	MG889395	This Study

Supplementary Table 3. Gene expression levels of *ThtAP3-1*, *ThtAP3-2a*, *ThtAP3-2b* and *ThtPI* for individual samples, averaged among three technical replicates and normalized to *ThtACTIN* and *ThtEF1α (Elongation Factor 1α)*.

		Gene Expression (2-ΔCt)			
Construct	Sample	*ThtAP3-1*	*ThtAP3-2a*	*TthAP3-2b*	*ThtPI*
None - Untreated	2.1	0.097508153	0.061568376	0.30319825	0.622005827
None - Untreated	2.2	0.093104841	0.024774504	0.31316611	0.685391402
None - Untreated	3.1	0.142102122	0.028261856	0.209981384	0.431769528
None - Untreated	3.2	0.08227892	0.033415625	0.224273852	0.448547704
None - Untreated	7.1	0.082851215	0.035566595	0.228457863	0.463294031
None - Untreated	7.2	0.13490353	0.0838139	0.488579984	0.768437591
TRV2-Empty	10.1	0.150031083	0.020099917	0.179659027	0.538368784
TRV2-Empty	10.2	0.185994689	0.077481731	0.75610928	0.970410231
TRV2-Empty	10.3	0.167240944	0.067140791	0.54841249	0.567752215
TRV2-ThtAP3-1	2.2	0.037637988	0.028524261	0.200036245	0.386444552
TRV2-ThtAP3-1	2.4	0.023601134	0.037464465	0.203297822	0.537747195
TRV2-ThtAP3-1	7.3	0.012618346	0.135685018	0.28029152	0.813191289
TRV2-ThtAP3-1	7.7	0.012809281	0.088388348	0.327598351	0.454809197
TRV2-ThtAP3-1	7.1	0.047805928	0.054535268	0.357661483	0.753493341
TRV2-ThtAP3-1	13.2	0.016232173	0.013808177	0.023492325	1.029302237
TRV2-ThtAP3-1	13.3	0.015643061	0.047093334	0.093104841	0.660516573
TRV2-ThtAP3-1	15.1	0.003988329	0.022929256	0.129109464	0.374576769
TRV2-ThtAP3-1	15.3	0.010464641	0.020880981	0.156945257	0.547779305
TRV2-ThtAP3-1	2.1	0.01845301	0.042886478	0.332171454	0.750019495
TRV2-ThtAP3-1	2.5	0.031070013	0.037899781	0.40007249	0.698177934
TRV2-ThtAP3-1	2.3	0.083331167	0.019214421	0.470304378	1.155352697
TRV2-ThtAP3-1	7.1	0.017039183	0.063445721	0.184496668	0.593231317
TRV2-ThtAP3-1	7.9	0.013415085	0.010284865	0.06410882	0.465439858
TRV2-ThtAP3-1	15.2	0.01064756	0.013888167	0.177185608	0.487452428
TRV2-ThtAP3-2a	1.1	0.146604369	0.136786713	0.409896999	0.764894847
TRV2-ThtAP3-2a	1.2	0.219658363	0.13030822	0.565134695	1.302846093
TRV2-ThtAP3-2a	1.4	0.19144475	0.203768083	0.629233186	0.61913818
TRV2-ThtAP3-2a	2.3	0.434772722	0.07492893	0.517034815	0.959264119

TRV2-ThtAP3-2a	2.4	0.294566785	0.059539875	0.542113435	0.860551437
TRV2-ThtAP3-2a	12.1	0.227930622	0.1129169	0.471937156	0.961483052
TRV2-ThtAP3-2a	12.2	0.22635619	0.135528359	0.848703971	1.529789694
TRV2-ThtAP3-2a	12.3	0.110977055	0.167821563	0.414181016	0.746561664
TRV2-ThtAP3-2a	1.5	0.463294031	0.225312616	1.479387509	2.345669898
TRV2-ThtAP3-2a	1.6	0.312804535	0.175150458	0.68380964	0.987372704
TRV2-ThtAP3-2b	7.1	0.345079338	0.033223165	0.067373886	0.531570642
TRV2-ThtAP3-2b	10.1	0.082374027	0.042149714	0.087676458	0.397308987
TRV2-ThtAP3-2b	20.5	0.289506304	0.010392357	0.010635267	0.564482202
TRV2-ThtAP3-2b	20.8	0.153360622	0.028922445	0.028989347	0.771996743

Supplementary Table 4. Sequence and location of putative AP3 binding sites in *Thalictrum thalictroides* B-class gene promoters, based on MORPHEUS analysis (with threshold score > 5). Bolded sequences denote the actual strand, sense (+) or antisense (-), for the putative binding site. MORPHEUS score and position of the binding site relative to the ATG start codon are listed.

**Locus**	**CArG box binding Site (strand)**	**Score**	**Position (Relative to ATG start codon)**
ThtAP3-1	CACCCACTTTGAGAA(+)<br>**TTCTCAAAGTGGGTG (-)**	5.1	-262
	**TCACTCAAGTAGAAA (+)**	6.5	-139
	TTTCCATTTTTAGAA(+)<br>**TTCTAAAAATGGAAA (-)**	11.9	-121
ThtAP3-2a	CTTCCTTATATAGAG(+)<br>**CTCTATATAAGGAAG (-)**	5.6	-91
ThtAP3-2b	TGTCCATTTGAAGAC (+)<br>**GTCTTCAAATGGACA (-)**	5.3	-293
	**TTCTTAAACTAGTAA (+)**	5.3	-151
	TTTCCTTTTATAGAA (+)<br>**TTCTATAAAAGGAAA (-)**	9.7	-78
ThtPI	TTGCTTCTTAAGTCT (+)<br>**AGACTTAAGAAGCAA (-)**	5.2	-340
